# Immune-enrichment of non-small cell lung cancer baseline biopsies for multiplex profiling define prognostic immune checkpoint combinations for patient stratification

**DOI:** 10.1186/s40425-019-0544-x

**Published:** 2019-03-28

**Authors:** Anne Monette, Derek Bergeron, Amira Ben Amor, Liliane Meunier, Christine Caron, Anne-Marie Mes-Masson, Nidhameddine Kchir, Kamel Hamzaoui, Igor Jurisica, Réjean Lapointe

**Affiliations:** 1 Institut du cancer de Montréal, Montréal, Québec Canada; 2 Centre de Recherche du Centre Hospitalier de l’Université de Montréal (CRCHUM), 900 rue St-Denis, Tour Viger, Room R10-432, Montréal, Québec H2X 0A9 Canada; 3 Département de Médecine, Faculté de Médecine, Université de Montréal, Montréal, Canada; 4 Medicine Faculty of Tunis, Department of Immunology and Histology, Tunis El Manar University, Tunis, Tunisia; 5 Department of Pathology, La Rabta Hospital of Tunis, Tunis, Tunisia; 6 Abderrahmen Mami Hospital, Homeostasis and cell immune dysfunction Research Unit, Ariana, Tunisia; 7 Krembil Research Institute, UHN, 60 Leonard Avenue, Toronto, Ontario M5T 0S8 Canada; 8 Institute of Neuroimmunology, Slovak Academy of Sciences, Bratislava, Slovak Republic

**Keywords:** Lung cancer, Baseline biopsies, Prognostic immune checkpoint combinations, Pan-cancer

## Abstract

**Background:**

Permanence of front-line management of lung cancer by immunotherapies requires predictive companion diagnostics identifying immune-checkpoints at baseline, challenged by the size and heterogeneity of biopsy specimens.

**Methods:**

An innovative, tumor heterogeneity reducing, immune-enriched tissue microarray was constructed from baseline biopsies, and multiplex immunofluorescence was used to profile 25 immune-checkpoints and immune-antigens.

**Results:**

Multiple immune-checkpoints were ranked, correlated with antigen presenting and cytotoxic effector lymphocyte activity, and were reduced with advancing disease. Immune-checkpoint combinations on TILs were associated with a marked survival advantage. Conserved combinations validated on more than 11,000 lung, breast, gastric and ovarian cancer patients demonstrate the feasibility of pan-cancer companion diagnostics.

**Conclusions:**

In this hypothesis-generating study, deepening our understanding of immune-checkpoint biology, comprehensive protein-protein interaction and pathway mapping revealed that redundant immune-checkpoint interactors associate with positive outcomes, providing new avenues for the deciphering of molecular mechanisms behind effects of immunotherapeutic agents targeting immune-checkpoints analyzed.

**Electronic supplementary material:**

The online version of this article (10.1186/s40425-019-0544-x) contains supplementary material, which is available to authorized users.

## Background

Lung cancer accounts for the majority of cancer-related deaths, with almost two million diagnosed globally each year [1], and non-small cell lung carcinoma (NSCLC) representing 83% of cases [2]. Though surgical resection is the preferred treatment modality, most patients are diagnosed at advanced, unresectable stages. The TNM staging system has historically been the most widely used predictor of NSCLC survival. Adenocarcinoma (ADC) and squamous-cell carcinoma (SCC) subtypes have differing prognostic and predictive profiles [3]. As such, pathologists are mandated to distinguish subtypes, regardless of size and quality of biospecimens, ahead of targeted and personalized therapies [4]. Advances in subtyping have brought into question the requirement for TNM [5], and recent studies demonstrate that use of immunohistochemistry (IHC) cocktails and bioinformatics [6, 7], provides comparable accuracy between poorly differentiated lung biopsies and large tumors [8, 9].

The ability of T cells to control cancers is now widely accepted. The use of the adaptive immune system as prognostic and predictive is becoming standardized from indisputable evidence of immunosurveillance [10], and the Immunoscore (IM) outperforming TNM staging [11]. Though tumor infiltrating lymphocytes (TIL) are associated with positive outcomes, their anti-tumor activity is curbed by immune checkpoints (ICP). ICP-blockade therapies showing broad efficacy in NSCLC patients relative to standard care are now front-line treatments [12]. Differential responses to treatments has prompted rapid FDA approval of PD-L1 companion diagnostic (CDx) assays, and measures are being taken to address its heterogeneity and assay discordance [13]. From vast clinical successes from PD-1/PD-L1 targeting, numerous additional ICPs are being investigated as combinatorial targets or CDx to control cancer [14], autoimmunity [15], and numerous infectious diseases [16]. Initially categorized as exhaustion markers of functionally impaired T cells, ICPs are expressed by tumor-reactive TILs sharing tumor-antigen specificities and T cell receptor (TCR) repertoires with circulating ICP expressing T cells [17], suggesting these may identify responders to immunotherapies.

Diagnosis and staging of NSCLC is commonly established from core needle biopsy and fine-needle aspiration, however the size and heterogeneity of these specimens does not permit use of standard IM or PD-L1 assays, creating a critical need for the development of biopsy-adaptable CDx. We constructed a tissue microarray (TMA) from immune-dense regions of core needle biopsies from a baseline NSCLC cohort, and used it to profile infiltrating immune cell (IIC) subsets, ICPs, proliferation, and effector T cell markers. We find combinations that efficiently stratify patients, and validate prognostic ICP-signatures on additional cohorts. We profile ICP coexpression dynamics and ICP linkage to clinical parameters and IIC subsets, map ICP-interactors and associated pathways, and define the most prognostic combinations able to guide blockade therapies using baseline biospecimens of all sizes.

## Methods

### Study design

ICP were profiled using 17 lung cancer cohorts from differing origins, and using different methods: 1) at the protein expression level on a TMA created from a baseline NSCLC cohort (*n* = 81) (Additional file 1: Table S1; La Rabta Hospital of Tunis, Tunis, Tunisia); 2) at the whole-tumor RNA level using RNA-Seq datasets from two NSCLC cohorts from the TCGA, the LUAD (*n* = 504) and LUSC (*n* = 494) (http://www.cbioportal.org); and 3) at the whole-tumor RNA level using microarray datasets from 14 NSCLC cohorts from the GEO, the EGA and TCGA (*n* = 2435) Kaplan-Meier Plotter (http://kmplot.com). Additional breast (*n* = 5143), gastric (*n* = 2183), and ovarian (*n* = 1816) cohort datasets were from Kaplan-Meier Plotter. Written and informed consent procedures were approved by the ethics review committees and were obtained from patients prior to the collection of specimens. Clinical patient data was randomly numbered for complete anonymity. Censoring of cohort patient data was from time of diagnosis to last follow-up or death.

### TMA construction

**Fig. 1:**
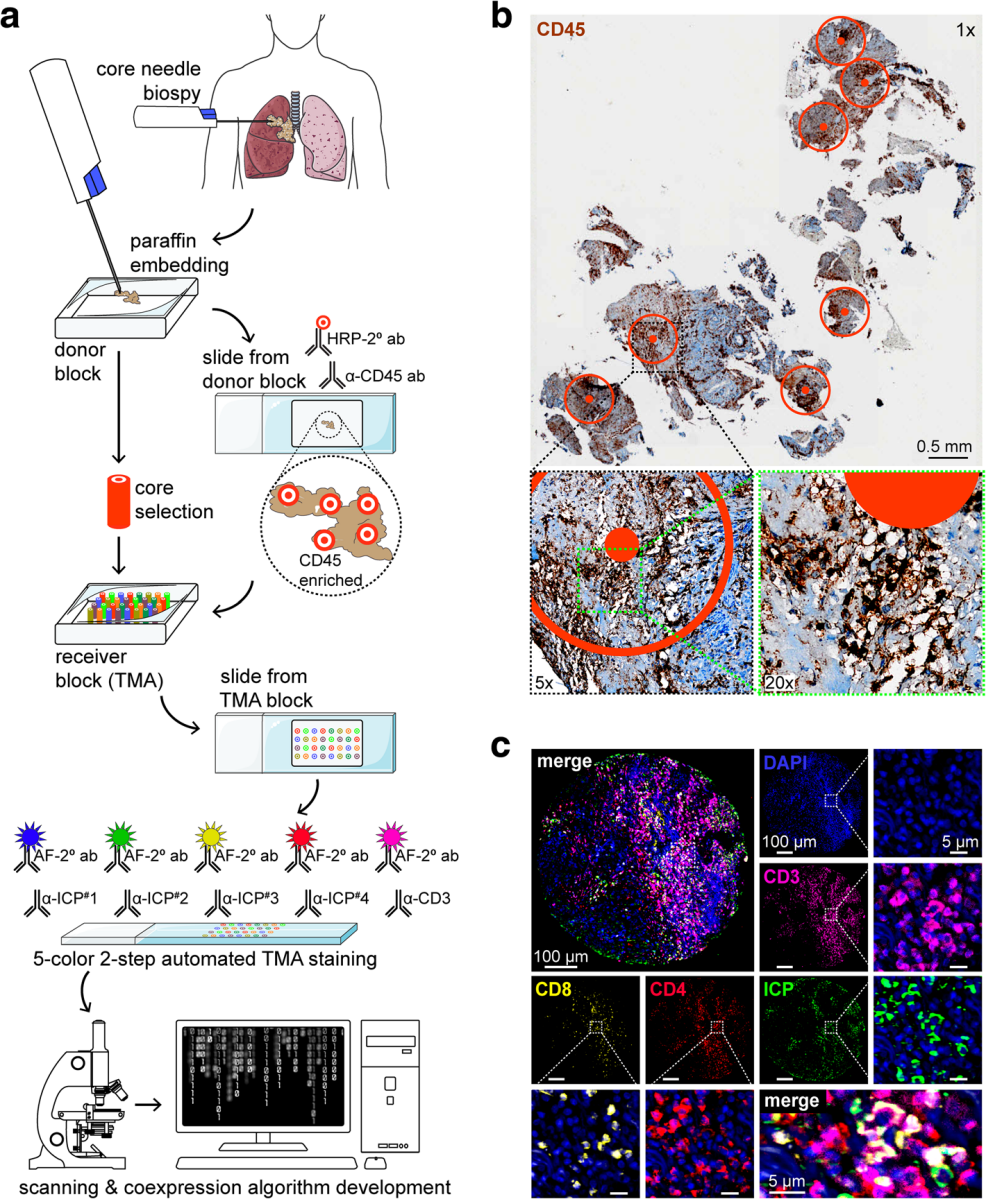
Creation and analysis of IIC-enriched biopsy-based NSCLC TMA. **a** Illustration depicting TMA creation workflow. Baseline biopsies from a NSCLC patient cohort (*n* = 81) were paraffin embedded, and cut sections were stained using α-CD45 to demarcate IIC-dense regions then selected for TMA construction using original blocks. Cut sections from resulting TMA were then stained using MP-IF panels targeting immune-related antigens including ICPs and IIC subsets. Slides were scanned to create super images permitting the development of algorithms computing antigens of interest and their colocalization for normalization (figure elements modified from Servier medical art). **b** Image representing α-CD45 IHC stained biopsies defining IIC-dense areas. **c** Example of MP-IF panels demonstrating α-ICP (green), α-CD3 (pink), α-CD4 (red), and α-CD8 (yellow) antibodies validated to surround DAPI-staining nuclei (blue). IIC-enriched core selection was performed by two different operators. TMA cores were randomized and TMAs were created by two operators. HRP, horseradish peroxidase; 2° ab, secondary antibody; AF, Alexa-Fluor dye; α, anti; μm, micron; mm, millimeter

An illustration of the TMA construction is provided in Fig. 1a. Four μm cuts made using a microtome (Leica Biosystems) from all biopsies were α-CD45 stained for IHC using the Benchmark XT automated stainer with CC1 antigen retrieval buffer (Ventana Medical Systems) for 1 h. Slides were incubated with α-CD45 (1:50) at 37 °C for 1 h, followed by the ultraView DAB detection kit and counterstaining with haematoxylin and bluing reagent (Ventana Medical Systems). Slides were scanned with an Olympus BX61VS microscope equipped with a VS110 slide scanner and 20x / 0.75 NA objective with a resolution of 0.3225 mm (Olympus). Images were exported and visualized using OlyVia image viewer software ver. 2.8 (Olympus) to identify CD45^+^ IIC-rich regions. Three to five IIC-rich regions of the biopsies were selected for 0.6 mm core transfer into the receiving TMA paraffin block using a TMArrayer (Pathology Devices). Paraffin blocks were kept at 4 °C until used for TMA construction. TMA cores were press-sealed into place after incubation at 50 °C for 10 min. The TMA was cooled at RT ON, and was chilled on ice ahead of being cut into 4 μm sections. Sections were floated onto 1 mm slides (Fisher Scientific), dried ON, and stored at 4 °C until stained.

### Multiplex-immunofluorescence

TMA sections were deparaffinized by incubation at 50 °C for 1 h prior to 5 min incubations in successive baths (3x xylene, 95, 90, 70, and 50% ethanol, dH_2_O). Antigen retrieval was performed using Target Retrieval Solution, Citrate pH 6 (DAKO) as recommended by the manufacturer. Protein Block (DAKO) was applied against non-specific staining for 40 min. Slides were rinsed with PBS before incubation with primary antibody mixtures diluted in Antibody Diluent (DAKO), 0.05% Tween 20 (Fisher Scientific) ON in a humidified chamber at 4 °C. Antibodies and their dilutions are in Additional file 1: Table S3. Following three 15 min PBS washes, slides were incubated with secondary antibody mixes for 1.5 h at RT (cross-adsorbed donkey α-rabbit, α-rat, or α-goat IgG (H&L) and/or goat α-mouse IgG1, IgG2a, IgG2b or IgGM specific secondary antibodies conjugated to Alexa-Fluors (405, 488, 594, 647 and 750) (ThermoFisher Scientific and Abcam) (1:250) Additional file 1: Table S3. Slides were washed with three 15 min incubations in PBS, and incubated in Sudan Black (1% in 70% ethanol) for 15 min. Slides were washed with dH_2_O for 5 min, and dried for 30 min before being set with ProLong gold antifade reagent (±DAPI) (ThermoFisher Scientific) under 0.17 mm coverslips (Fisher Scientific). Primary antibodies were individually detected by donkey α-host IgG (H&L) Alexa-Fluor 594 antibodies, and images were acquired using a Zeiss Axio Observer Z1 automated microscope equipped with a Plan-Apochromat 20x / 0.8 NA objective, a Zeiss HRm Axiocam and LED pulsed light illumination (Additional file 1: Figure S1d). Fluorescence minus one controls were used for potential fluorescence bleed-through between detection channels. In other control experiments, primary antibodies: 1) were not added, 2) were detected by alternative secondary antibodies, 3) were tested on a TMA containing 14 cancer cell lines (e.g., prostate, breast, ovarian, kidney, cervical cancer cells and Jurkat), and 4) were replaced with isotype control antibodies (MOPC-31C, G155–178, MPC-11) (BD Pharminogen). MP-IF stained slides were scanned using an Olympus BX61VS microscope housing a BrightLine Sedat filter set (Semrock) optimized for DAPI, FITC, TRITC, Cy5 & Cy7, and equipped with a 20x / 0.75 NA objective with a resolution of 0.3225 mm and a VS110 slide scanner running FW-AS software (Olympus) that stitches individual images to build high resolution .vsi images.

### Image analysis

High resolution images were imported into Visiomorph software (Visiopharm), where cores were identified and linked to patient numbers using an Array-Imager module. Using fluorescence intensity thresholding, algorithms were designed to define a region of interest (ROI) and calculate total core area, which was further trained to eliminate holes within tissues to correct for actual tissue-occupying areas (Additional file 1: Figure S1f). Two independent operators used fluorescence intensity thresholding and size exclusions to create algorithms generating labels counting cells positive for biomarkers. Single marker labeling and co-labeling of dual, triple, and quadruple colocalizing markers were performed in the same way. For co-labeling, labels created for counting cells positive for multiple biomarkers were determined using the same thresholds used to identify and count single marker labeling cells. Created co-labels were also verified as accurately staining immune cells by two independent operators. Labels identifying markers were adjusted for IIC sizes, and were centered on DAPI staining when present in panels. Baseline fluorescence thresholds assigned for minimal signal to noise ratios determining positivity were used to calculate MFIs. Counts of algorithm-determined labels on cores were validated to reflect visual operator counts. Inter-rating correlations from algorithms created by independent operators was assessed to be > 75%. Each single or multi-marker label counts (e.g., totaling up to 15 marker permutations for each individual 5-color panel in case of DAPI + 4 markers) of individual cores were automated to be reassigned to patient ID numbers, and were then log-transformed and normalized to core size, prior to being merged with the clinical data for averaging of replicate core values, resulting in data from 73 patients for further analyses from .csv data file exports. High (hi) and low (lo) values were defined as being above or below mean ± SEM. Receiver operative characteristic (ROC) curves (SPSS software v.23, IBM), were used to validate that selected cutoff values corresponded to the best sensitivity and specificity any given marker. ICPs having inter-patient variability were found from a second method of analysis applied whereby values from individual cores were not averaged.

### Statistical analysis

Power analysis determined that our retrospective biomarker study based on overall patient survival required a minimal sample size of *n* = 62 to reach a power of 0.80 at α = 0.05 (two-tailed) (G*Power ver. 3.1.9.2; Universitat Düsseldorf, Germany). Prism 6 ver. 6.01 (GraphPad) and SPSS software packages were used for statistical analysis of biomarkers with patient data. Log-rank (Mantel-Cox) tests with log-rank HR were used for K-M. A student’s t test was used to compare two groups, and two-way ANOVA (with Tukey’s or Bonferroni’s multiple-comparisons tests) was used for multiple comparisons. Pearson correlation coefficients were calculated with two-tailed *P* values with 95% confidence intervals. *P*-values of less than 0.05 were considered to indicate a statistically significant difference. R with a collection of libraries was used for additional statistical correlation, linear regression, variance and clustering analysis, patient clinical characteristics and biomarker expression value relationships analyses. Here, expression values were log transformed towards a Gaussian distribution. Linear regression matrices were computed using the R glm function. Link functions were adapted phenotype distribution type (binomial, Gaussian, Poisson) for model compatibilities for explorations of relationships between biomarkers and clinical data. K-M calculations, cox model *p*-values and HR were validated using a survival model coupling survival status and months of survival post biopsy. PCA was used for coexpression analysis. Cumulative correlations for the expression of each ICP (and CD3-ICP) were calculated from their respective correlation matrices.

### Prognostic signature validation and gene expression analysis

Kaplan Meier plotter was used to validate the prognostic value of the ICP signature, and to assess ICP gene expression modulation between tumors and normal tissues. Gene ID symbols were mapped to Affymetrix probes from GEO, EGA and TCGA datasets, and their mean expression was used to assess OS. For K-M, default settings were used with auto select best cutoff and best specific probes (JetSet probes). The 2017 version of Kaplan Meier plotter contains information on 54,675 genes for survival, including 2437 lung, 5143 breast, 1065 gastric, and 1816 ovarian cancer patients with mean follow-up times of 49, 69, 33, and 40 months, respectively. Multigene classifier function using default settings from KM-plotter was used to run the analysis on all ICPs simultaneously, where global ICP coexpression represents combined prognostic effects of all ICPs investigated in this study.

### Protein-protein interaction network and pathway enrichment analysis

Identified biomarkers were subjected to comprehensive pathway enrichment analysis using pathDIP ver. 2.5 (http://ophid.utoronto.ca/pathDIP) (Additional files 2 and 3). Default settings were used, with extended pathway associations (combining literature curated core pathways with associations predicted using physical protein interactions with minimum confidence levels of 0.99). Lists were also used to retrieve physical protein interactions and explore biologically relevant links. IID ver. 2016–03 (http://ophid.utoronto.ca/iid) was used to map identified biomarkers to proteins and retrieve their interacting partners. Default settings were used, and interactions among partners of query proteins, source information (detection methods, PubMed IDs, reporting databases), and tissue information (presence/absence of interactions in selected tissues) were included. Corresponding networks were visualized using NAViGaTOR ver. 3 (http://ophid.utoronto.ca/navigator) (Additional file 4). Word-cloud analysis was performed using Wordle software ver. 2014 (http://www.wordle.net).

## Results

### Creation and analysis of immune cell-enriched tissue microarray

We aimed to develop a standardizable, immune-based, prognostic scoring method for biopsies. To reduce tumoral heterogeneity, a CD45-enriched TMA was constructed from baseline biopsies from a NSCLC cohort (Additional file 1: Tables S1 and S2). Figure 1a illustrates the construction of the TMA. Ahead of construction, nine random biopsy sections where stained for immunofluorescence (IF) using DAPI, α-CD45 and α-cytokeratin; verifying these for epithelial cancer and IIC-densities (Additional file 1: Figure S1a). Cut sections from all biopsies were then stained for IHC using α-CD45, defining IIC dense regions selected for TMA construction (Fig. 1b). IIC density of biopsies did not correlate with clinical parameters (*P* > 0.416) (Additional file 1: Figure S1b) or overall survival (OS) (*P* = 0.7880) (Additional file 1: Figure S1c). All antibodies were validated independently (Additional file 1: Figure S1d and e), and TMAs were stained with five-color multiplex-IF (MP-IF) panels using a two-step, semi-automated method (Fig. 1a and c). Algorithms calculated core areas to normalize labels identifying size- and fluorescence intensity-gated, colocalizing IICs and ICPs (Additional file 1: Figure S1f).

### Proliferating effector TIL and TIL-B densities correlate with improved survival

**Fig. 2:**
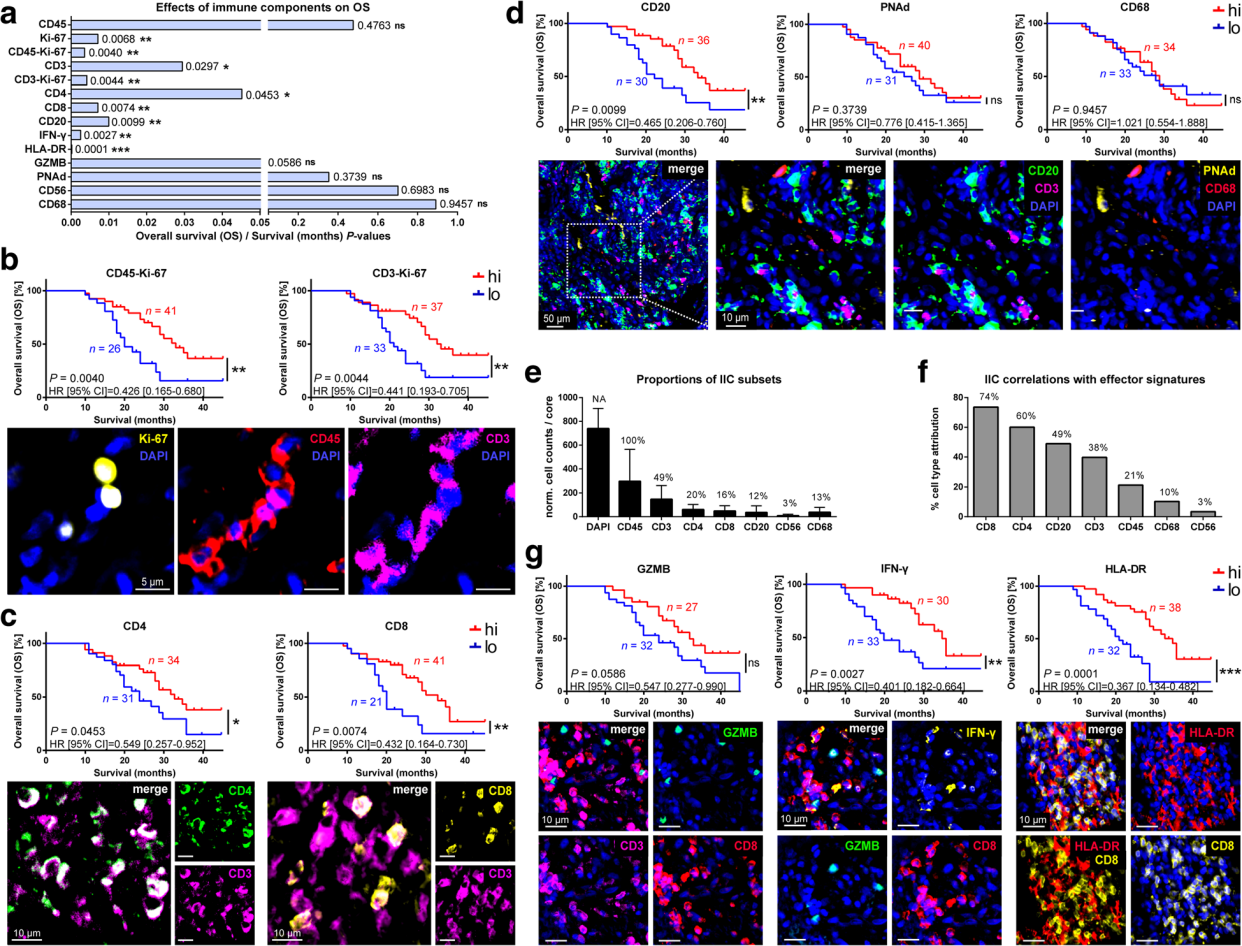
Highly proliferating, effector TIL and TIL-B densities are linked to positive prognostic of NSCLC patients. **a** Summarizing graph of *P*-values generated from K-M survival analyses of markers applied to IIC-enriched biopsy TMA, where significance indicates positive associations of IIC subsets, and proliferation and effector molecules with OS. **b** K-M curves (top) from Ki-67 co-labeling with CD45^+^ IICs or CD3^+^ TILs on TMA, and representative close-up IF images from cores (bottom) demonstrating co-labeling on cells. **c** K-M curves (top) from CD4^+^ and CD8^+^ TILs on TMA, with representative close-up IF images from cores (bottom) demonstrating their co-labeling CD3^+^ TILs. **d** K-M curves (top) from CD20^+^ TIL-Bs, PNAd^+^ HEV, and CD68^+^ TAMs, with representative close-up IF images from cores (bottom). **e** Graph of average proportions of IIC subsets relative cell count (DAPI), where percentages represent IIC subset abundance relative to CD45^+^ IICs. Percentages are relative to CD45 content, and error bars represent mean ± s.d.. **f** Graph of correlations between IIC subsets and quantified effector molecules (IFN-γ, GZMB, HLA-DR). Percentages represent IIC subset attribution to effector molecule expression, as calculated from proportions of individual IIC subsets infiltrating cores expressing effector molecules. **g** K-M curves (top) of GZMB, IFN-γ, and HLA-DR effector markers, with representative close-up IF images from cores (bottom) of these markers and TILs. The number of patients (*n*) for each group is given on K-M curves, and remainder are in Additional file 1: Figure S2b. Algorithm design, normalization and analyses were performed by two independent operators. Norm., normalized; hi, high marker expression, lo, low marker expression; μm, micron; *P*, Log-rank test; ns, not significant; * *P* < 0.05; ** *P* < 0.01; *** *P* < 0.001; HR, hazard ratio (Log-rank); CI, confidence interval of ratio; NA, not applicable

To determine whether IIC subsets and activation markers could predict OS, TMAs were stained with MP-IF panels labeling CD45^+^ leukocytes; CD3^+^, CD4^+^, and CD8^+^ T cells; CD20^+^ B cells; CD56^+^ natural killer (NK) cells; CD68^+^ macrophages; proliferating cells (Ki-67^+^); and activation and cytotoxic markers (human leukocyte antigen-DR, HLA-DR^+^; granzyme B, GZMB^+^; interferon-gamma, IFN-γ^+^). IIC densities of TMA cores had Gaussian distribution (Additional file 1: Figure S2a). Kaplan-Meier survival analyses (K-M) demonstrated that CD45 density did not correlate with OS (*P* = 0.4763) (Fig. 2a and Additional file 1: Figure S2b), as expected from its demarcating all IIC subsets having differential effects on prognoses. Ki-67 was associated with positive OS (*P* = 0.0068) (Fig. 2a and Additional file 1: Figure S2b), contrary to Ki-67 in cancer-centric studies [18], and attributable to an IIC-enriched TMA. OS was associated with CD45-Ki-67 co-labeling cells (*P* = 0.0040) (Fig. 2a and b). The same was observed for TILs, where association of CD3 with OS was enhanced by Ki-67 co-labeling (*P* = 0.0297 to *P* = 0.0044) (Fig. 2a and b, and Additional file 1: Figure S2b). CD4^+^ TILs were modestly associated with OS (*P* = 0.0453) (Fig. 2a and c), likely due to this mixed population having differential effects on prognosis [19]. CD8^+^ TILs strongly associated with OS (*P* = 0.0074) (Fig. 2a and c) [20].

Effector CD8^+^ TILs mediate anti-tumor immunity in cooperation with tumor infiltrating CD20^+^ B cells (TIL-B) [21]. B cells serve as antigen presenting cells (APC), and secrete cytokines and chemokines causing IIC tumor homing across high endothelial venules (HEV) to induce tertiary lymphoid structures driving anti-tumor responses and long-term immunity [22, 23]. CD20^+^ TIL-Bs were significantly associated with OS (*P* = 0.0099) (Fig. 2a and d). A HEV marker, Peripheral node addressin (PNAd), showed no association with OS (*P* = 0.3739) (Fig. 2a and d). CD68^+^ tumor-associated macrophages (TAMs) were also not associated with OS (*P* = 0.9457) (Fig. 2a and d), as CD68 cannot distinguish M1 and M2 subsets having countering effects on prognosis [24]. Likewise, CD56^+^ NK cells had no effects on OS (*P* = 0.6983) (Fig. 2a). We compared proportions of IIC subtypes to assess whether their linkage with OS reflected density. We averaged 742 ± 163 cells per TMA core [25], with 40 ± 25% CD45^+^ IICs of all DAPI^+^ cells. Though representing a lower proportion of CD45^+^ IICs (normalized to 100%), CD20^+^ TIL-Bs (representing 12 ± 5% of all CD45^+^ IICs) had greater association with OS (*P* = 0.0089) than CD3^+^ TILs (representing 49 ± 11% of all CD45^+^ IICs; *P* = 0.0297) (Fig. 2a and e).

Cytotoxic and immune stimulation markers were investigated. Correlative studies between expression of effector markers (IFN-γ, GZMB, HLA-DR), and IIC subset infiltration of patient cores were used to demonstrated that expression of effector markers could be associated with the presence of CD8^+^, CD4^+^ and CD20^+^ IICs (Fig. 2f). IFN-γ (*P* = 0.0027) and HLA-DR (*P* = 0.0001) were positively associated with OS (Fig. 2a and e). IFN-γ marks adaptive immune activation, and is central to anti-tumor immunity [26], and absence of HLA-DR is associated with metastasis [27]. IFN-γ localized to plasma membranes and periplasmic bursts of CD8^+^ TILs, and to nuclei of both TILs and epithelial cells (Additional file 1: Figure S1e), possibly explained by its rapid cellular export and nuclear localization signal [28]. GZMB and HLA-DR staining was typical, but rarely evident on TILs (Fig. 2g). HLA-DR is expressed by APCs [29], perhaps explaining it labeling cells neighboring CD8^+^ TILs. As prognostic factor for NSCLC, HLA-DR has been shown to identify M1 CD68^+^ TAMs [30]. GZMB labeled small cells, and is expressed by B cells, mast cells, keratinocytes, and basophils [31]. Altogether, these results demonstrate that proliferating Ki-67^+^ IIC; CD3^+^, CD8^+^, and CD4^+^ TILs; CD20^+^ TIL-Bs; and HLA-DR and IFN-γ are positive prognostic markers for NSCLC patients.

### NSCLC survival correlates with increased expression of ICP on TIL

IFN-γ expression by activated TILs increases PD-L1 expression [32]. IFN-γ is also correlated with the expression of other ICPs, including BTLA [33], TIM-3 [34], LAG-3 [35], and PD-1 [36]. Since ICPs are expressed by various cell types, their usage as mono-CDx will lead to assay inconsistencies exemplified by PD-L1 [37]. Indeed, on our TMA, certain ICPs labeled numerous cells types (PD-L1, TIM-3, TIGIT, LAIR-1, CD73), whereas others almost exclusively labeled TILs (BTLA, LAG-3, PD-1, CD39, 2B4, CD57, CD26, CLTA-4) (Additional file 1: Figure S3a to e). Despite this, principal component analysis (PCA) demonstrated that relative to patients, tight clustering of ICPs and cognate CD3-ICPs indicated that they were mostly labeling TILs, and not other cells of the tumor microenvironment (Additional file 1: Figure S3f).

**Fig. 3:**
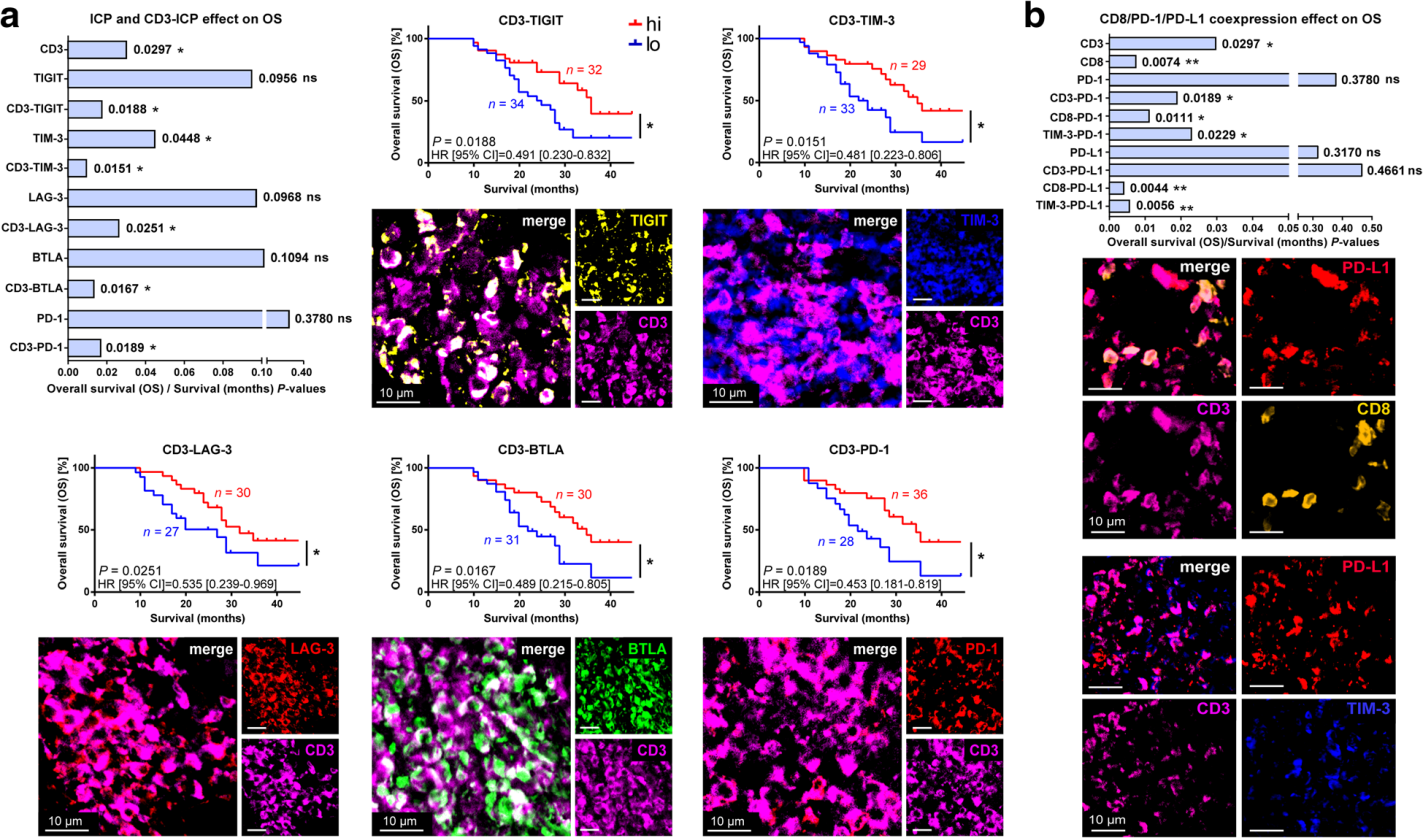
Effects of ICP expression on NSCLC patients. **a** Summarizing graph of *P*-values generated from K-M survival analyses of IIC-enriched TMA, where significance indicates positive associations ICP and CD3-ICP co-labeling cells with OS (top left). K-M curves and representative close-up IF images from cores (right and bottom) of CD3 dense core areas, demonstrating colocalization between CD3 and TIGIT, TIM-3, LAG-3, BTLA, or PD-1. **b** Summarizing graph of *P*-values generated from K-M survival analyses of IIC-enriched TMA, where significance indicates positive associations of combinations of CD3, CD8, PD-1, PD-L1 and TIM-3 with OS (top). Representative close-up IF images from cores (bottom) of CD3 dense core areas, demonstrating colocalization between these antigens. The number of patients (*n*) for each group is given on K-M curves, and remainders are (high and low, respectively): CD3 *n* = 34hi, 32lo; CD8 *n* = 41hi, 21lo; TIGIT *n* = 26hi, 38lo, TIM-3 *n* = 21hi, 26lo, LAG-3 *n* = 29hi, 33lo, BTLA *n* = 30hi, 30lo, PD-1 *n* = 36hi, 30lo, CD3-PD-1 *n* = 29hi, 24lo; CD8-PD-1 *n* = 36hi, 32lo; TIM-3-PD-1 *n* = 34hi, 30lo; PD-L1 *n* = 25hi, 33lo; CD3-PD-L1 *n* = 18hi, 30lo; CD8-PD-L1 *n* = 19hi, 24lo; TIM-3-PD-L1 *n* = 25hi, 34lo. Algorithm design, normalization and analyses were performed by two independent operators. hi, high marker expression, lo, low marker expression; μm, micron; merge, merge of all IF channels; *P*, Log-rank test; ns, not significant (implied when no asterisk is present); * *P* < 0.05; ** *P* < 0.01; HR, hazard ratio (Log-rank), CI, confidence interval of ratio

The only ICP associated with positive OS independently of TILs was TIM-3 (*P* = 0.0448), and this was augmented by it co-labeling CD3^+^ TILs (*P* = 0.0151) (Fig. 3a). Association with OS for other ICPs was only met by their co-labeling CD3^+^ TILs: CD3-TIGIT (*P* = 0.0188), CD3-LAG-3 (*P* = 0.0251), CD3-BTLA (*P* = 0.0167), and CD3-PD-1 (*P* = 0.0189) (Fig. 3a). While mean fluorescence intensities (MFI) of ICPs or all other markers tested showed no association with OS, some correlated with clinicopathological characteristics (Additional file 1: Table S4).

A refined analysis of PD-1 and PD-L1 on TILs was performed due to their importance as immunotherapeutic targets and CDx, and in light of recent clinical developments including FDA approved CDx assay for PD-L1 on IICs (SP142; Roche) and standardized Halioseek PD-L1/CD8 assay (HalioDx). We observe that co-labeling of CD8^+^ TILs with both PD-1 (*P* = 0.0111) and PD-L1 (*P* = 0.0044) increased positive association with OS (Fig. 3b). TIM-3 was also observed to provide survival advantages to PD-1 and PD-L1 (Fig. 3b).

A valuable aspect of this study was testing effects of ICPs on OS by diverging methods yielding negligible overall results, but providing caution for use of certain ICPs as CDx. Using the first method reported, we averaged ICPs from individual patient cores, while in the second method, we treated cores as if they were individuals themselves. Only three ICPs showed discrepancies using the second method, where PD-1 (*P* = 0.0121), CD3-PD-L1 (*P* = 0.0155), CD26 (*P* = 0.0052), and CD3-CD26 (*P* = 0.0017) were positively associated with OS, but CD3-TIGIT was not (*P* = 0.4830). This indicates that expression of these ICPs is ill-conserved throughout the tumor, and are thus less suitable as CDx candidates.

### Global ICP expression is independent of immune density and provides a pan-cancer survival advantage

**Fig. 4:**
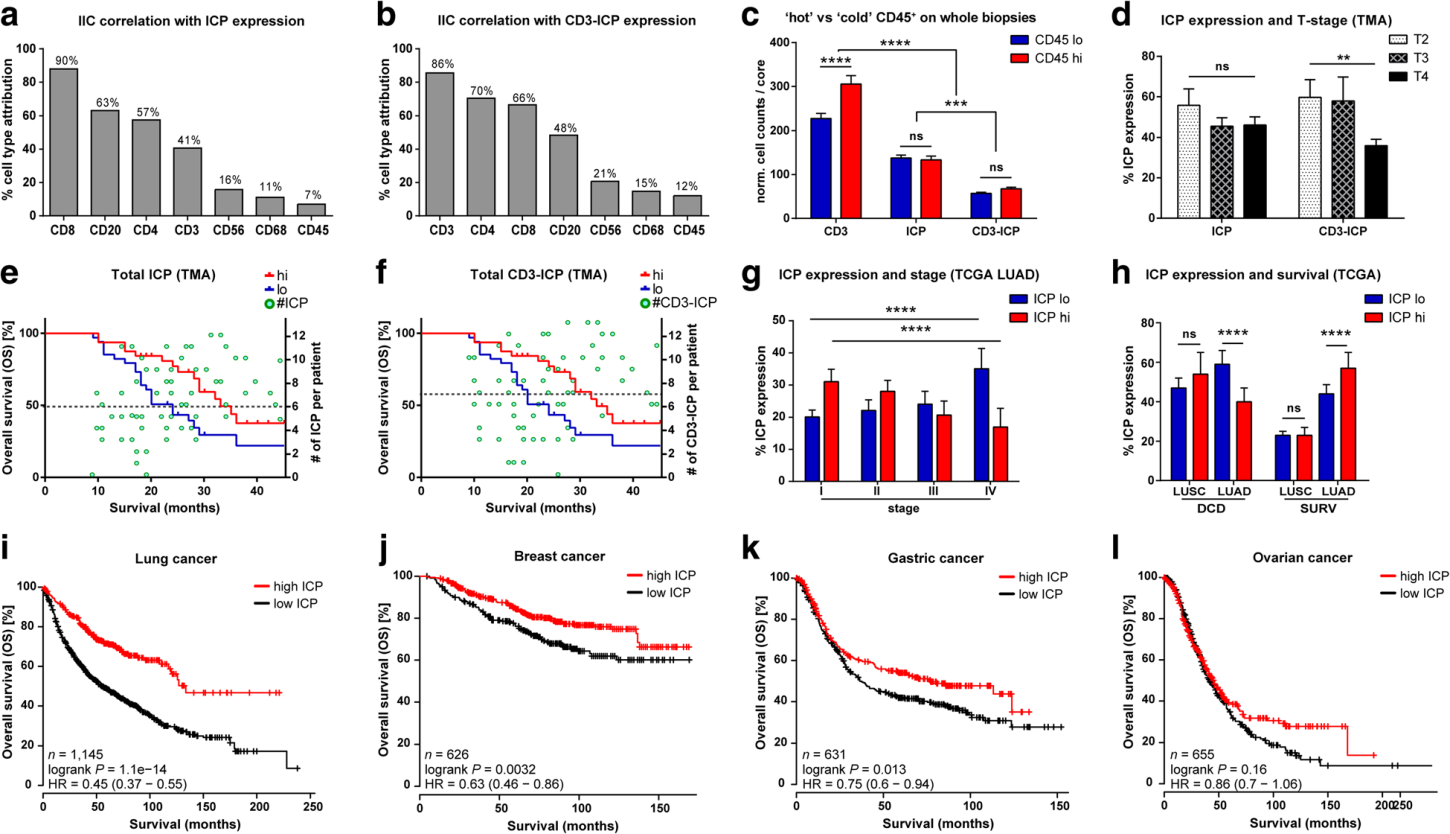
Effects of IIC density on global ICP expression and validation of global ICP prognostic effects on various cancers. **a**-**b** Graphs demonstrating correlations between TMA IIC subsets and (**a**) ICP or (**b**) CD3-ICP expression (%, IIC subset attribution; *n* = 73). Percentages represent IIC subset attribution to ICP or CD3-ICP expression, as calculated from proportions of individual IIC subsets infiltrating cores expressing ICPs or CD3-ICPs. **(c)** Graph demonstrating correlation between IIC-density of biopsies and CD3^+^ TILs, ICPs and CD3-ICPs. Two-way ANOVA with Bonferroni’s multiple comparisons test; *n* = 73, CD3, *P* < 0.0001; ICP vs CD3-ICP *P* = 0.005; F = 12.06, df = 1/219; error bars represent mean ± s.e.m.. **d** Graph demonstrating correlation of advancing T-stages with ICP expression (T2, *n* = 206; T3, *n* = 106; T4, *n* = 511) and CD3-ICPs (T2, *n* = 199; T3, *n* = 120; T4, *n* = 496) expression on TMA (two-way ANOVA with Tukey’s multiple-comparison; CD3-ICP T2 vs T4, F = 2.97, df = 2/1632, *P* = 0.0085; error bars represent mean ± s.e.m.). **e** K-M curve of total TMA ICP (*P* = 0.0273, HR [95% CI] = 0.514 [0.248–0.883], *n* = 32hi, *n* = 34lo) overlaid with number of ICP/patient relative to survival in months (green circles and right axis; dotted line, high vs low); linear regression of overlay F = 9.41, df = 1/62, *P* = 0.0032, R^2^ = 0.132. **f** K-M curve of total TMA CD3-ICP (*P* = 0.0472, HR [95% CI] = 0.546 [0.270–0.952], *n* = 30hi, *n* = 36lo) overlaid with number of CD3-ICP/patient relative to survival in months (green circles and right axis; dotted line, high vs low); linear regression of overlay F = 5.56, df = 1/63, *P* = 0.0215, R^2^ = 0.081. **g** Graph demonstrating correlation of advancing stage with ICP expression levels from LUAD dataset. Two-way ANOVA with Bonferroni’s multiple comparisons test, Stages I, *n* = 274; II, *n* = 121; III, *n* = 81; IV, *n* = 26, where stages I vs IV from both ICP hi or lo are *P* < 0.0001, F = 9.78, df = 3/996; error bars represent mean ± s.d.. **h** Graph demonstrating correlation of survival with ICP expression from TCGA LUAD and LUSC datasets. Two-way ANOVA with Bonferroni’s multiple comparisons test, *P* < 0.0001, F = 29.94, df = 1/828; ICP DCD, *n* = 172hi, 254lo; ICP SURV, *n* = 228hi, 178lo; error bars represent mean ± s.d.. **a**-**h** Algorithm design, normalization and analyses were performed by two independent operators. **i**-**l** K-M plots validating effects of global ICP expression on new cohorts of **(i)** NSCLC (*n* = 783hi, 362lo), (**j**) breast (*n* = 386hi, 240lo), (**k**) gastric (*n* = 265hi, 366lo), and **(l)** ovarian (*n* = 275hi, 380lo) cancer patients. Two-way ANOVA with Tukey’s post-test; norm., normalized; *n*, number of patients; SURV, surviving; DCD, deceased; *P*, Log-rank test; ns, not significant; ** *P* < 0.01; *** *P* < 0.001; **** *P* < 0.0001; HR, hazard ratio (Log-rank), CI, confidence interval of ratio

In correlative analyses between global ICP or CD3-ICP expression and IIC subsets, IIC subset infiltration of patient cores were used to demonstrated that expression of ICPs and CD3-ICPs effector markers could be most associated with the presence of CD8^+^, CD20^+^ and CD4^+^ IIC subsets (Fig. 4a and b). We tested whether the IIC-density of biopsies influenced CD3 and ICP distributions. CD3^+^ TILs were highly correlated with CD45^+^ IICs (*P* < 0.0001, *r* = 0.3428), but global ICP expression was not (Fig. 4c), with the exception of CD3-PD-1, CD3-PD-L1, CD3-BTLA and CD3-LAG-3 (Additional file 1: Table S5). This also supports that ICPs are not uniquely expressed by TILs (ICP vs CD3-ICP; *P* < 0.001) (Fig. 4c and Additional file 1: Figure S3a to e) [38, 39]. ICPs correlating with CD3 were BTLA, LAG-3, TIM-3 and CD26, and CD73 and CD3-CD73 correlated with the ADC subtype [40] (Additional file 1: Table S5). Despite their clear effects on outcomes (Additional file 1: Figure S4), there was no correlation between treatments and ICP expression. We also observed that CD3-ICPs were inversely correlated with tumor size and extent (Fig. 4d and Additional file 1: Table S5). K-M performed using global expression of ICP or CD3-ICP revealed that both positively correlated with OS (Fig. 4e and f), and global CD3-ICP expression also correlated with female gender (*P* = 0.0321, *r* = 0.0701).

Correlation studies relating IIC subtypes and other markers to clinicopathological characteristics were also performed. CD4, CD8, CD68 and IFN-γ inversely correlated with female gender (*P* < 0.0315, *r* = − 0.334), whereas HLA-DR and PNAd were positively correlated with it (*P* < 0.0469, *r* = 0.046). CD3 was inversely correlated with smoking (*P* = 0.0385, *r* = − 0.350), whereas PNAd was positively correlated with it (*P* = 0.0498, *r* = 0.606). CD20 and GZMB were inversely correlated with metastasis (*P* < 0.0370, *r* = − 0.333) (Additional file 1: Table S5).

To validate our findings on ICPs, we used the TCGA LUAD and LUSC RNA-Seq datasets. As observed from TMA analyses, advanced cancer patients and those deceased both had lower ICP expression (Fig. 4g and h). Despite background noise from these whole-tumor RNA datasets, eight ADC patient ICPs were associated with positive OS (Additional file 1: Table S6). Additional cohorts from the Gene Expression Omnibus (GEO), TCGA and European Genome-phenome Archive (EGA) validated this finding for ADC patients (*P* = 4.4e-05) (Additional file 1: Figure S5), and grouped analyses confirmed that global ICP coexpression benefited NSCLC patients regardless of subtype (*P* = 1.1e-14) (Fig. 4i). Global ICP coexpression was also positively associated with OS for breast (*P* = 3.2e-03) and gastric (*P* = 1.3e-02), but not ovarian cancers (*P* = 1.6e-01), despite an observable trend (Fig. 4j and l and Additional file 1: Table S7). These analyses also demonstrated a commonality of ICP expression in NSCLC and breast tumors relative to normal tissues (Additional file 1: Table S8). To validate the utility CDx profiling ICP on TILs, K-M was performed on ICP groups associated with OS or increased in expression, revealing that their prognostic value was maintained when coexpressing with CD4 or CD8 (Additional file 1: Table S9). These datasets also used to validate prognostic associations and increased expression of IIC subsets and T cell activation markers (Additional file 1: Table S10). Chromosomal locations of ICPs suggested that transcriptional regulation from common promoters is unlikely (Additional file 1: Table S11). Altogether, these results demonstrate that global ICP coexpression augments survival from different cancers, and their correlation with CD3^+^ TILs supports the development of multiplex CDx. Furthermore, since overall ICP expression was independent of IIC density, even patients with low infiltration may benefit from precision ICP-blockade therapies.

### ICP combinations on TIL are associated with increased NSCLC survival

**Fig. 5:**
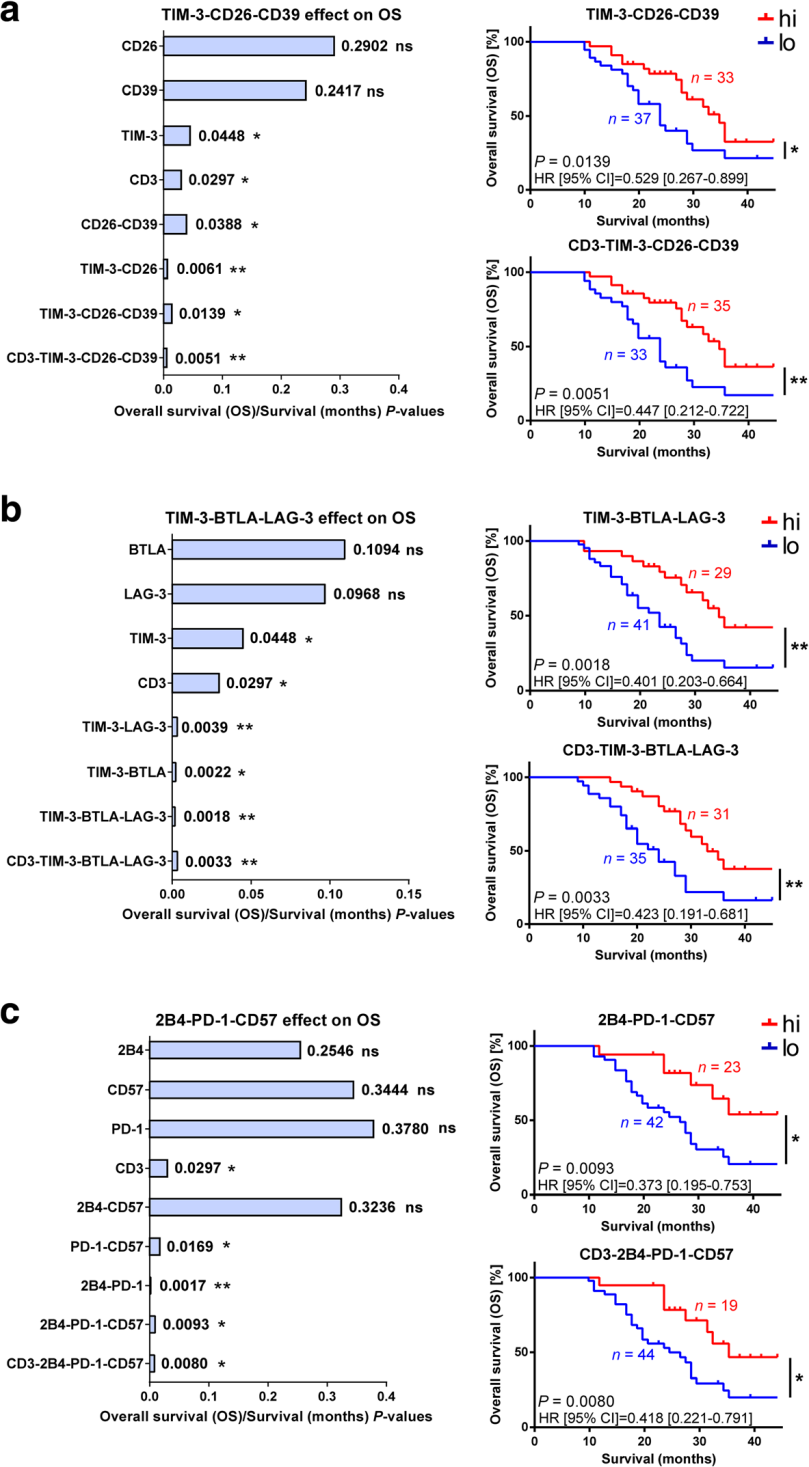
MP-IF panels for ICP combinations stratifying NSCLC patients. **a**-**c** Summarizing graph of *P*-values generated from K-M survival analyses (left), of ICP alone, and in combination with each other and with CD3 TILs, where significance indicates positive associations of combinations with OS. From top to bottom, panels interrogate combinations of CD3^+^ TILs and ICPs (**a**) TIM-3, CD26 and CD39, (**b**) TIM-3, BTLA and LAG-3, and (**c**) 2B4, PD-1, and CD57. K-M plots (right) illustrate similarities of curves of ICP combinations ± CD3 co-labeling. The number of patients (*n*) for each group is given on K-M curves, and others are either previously reported in Fig. 3, or are (high and low, respectively): CD26 *n* = 34hi, 32lo, CD39 *n* = 32hi, 26lo, CD26-CD39 *n* = 37hi, 35lo, TIM-3-CD26 *n* = 35hi, 37lo, TIM-3-LAG-3 *n* = 36hi, 32lo, TIM-3-BTLA *n* = 39hi, 31lo, 2B4 *n* = 31hi, 33lo, CD57 *n* = 29hi, 34lo, 2B4-CD57 *n* = 30hi, 35lo, PD-1-CD57 *n* = 27hi, 38lo, and 2B4-PD-1 *n* = 24hi, 44lo; associated confidence intervals are listed in Additional file 1: Table S12. Algorithm design, normalization and analyses were performed by two independent operators. Representative images of CD3-ICP colocalization-dense core areas can be found in Additional file 1: Figure S6. hi, high marker expression, lo, low marker expression; *P*, Log-rank test; ns, not significant; * *P* < 0.05; ** *P* < 0.01; HR, hazard ratio (Log-rank); CI, confidence interval of ratio

Using TMAs, we assessed minimal ICP combinations on TILs maximizing prognostic value (Additional file 1: Table S12). Indeed, the TIM-3/CD26/CD39 combination had a stronger association with OS than these did independently (*P* = 0.0139), and was superior when co-labeling with CD3 (*P* = 0.0051) (Fig. 5a). The positive effect on OS was maintained with ICPs and CD3-ICPs co-labeling for TIM-3/BTLA/LAG-3 combinations (*P* = 0.0018 to *P* = 0.0033), as it was for the 2B4/PD-1/CD57 combination (Fig. 5b and c). As supported by imaging (Additional file 1: Figure S6), comparisons of ICP and CD3-ICP K-M curves validated that these ICP combinations were specifically labeling TILs, and that the difference in prognostic association using duplex or triplex ICP panels was dependent on ICP combinations.

The feasibility of stratifying patients by adding individual ICP values instead of using ICP-colocalization values was also validated (e.g., TIM-3 + LAG-3, *P* = 0.0016; TIM-3 + BTLA, *P* = 0.0022; TIM-3 + BTLA+LAG-3, *P* = 0.0099), indicating that similar results could be attained from sequential IHC methods. However, our simplified method has less potential for antibody cross-reactions, loss of antigen and tissue integrity from harsh chemical treatments, loss of colocalization from permanent stains masking subsequent antigens, or potent spectral overlap of fluorescent signals requiring unmixing [41]. Altogether, these results demonstrate that the simultaneous detection of multiple ICPs on TILs using MP-IF panels efficiently stratifies NSCLC patients.

### Prognostic ICP groups are conserved across RNA and protein

**Fig. 6:**
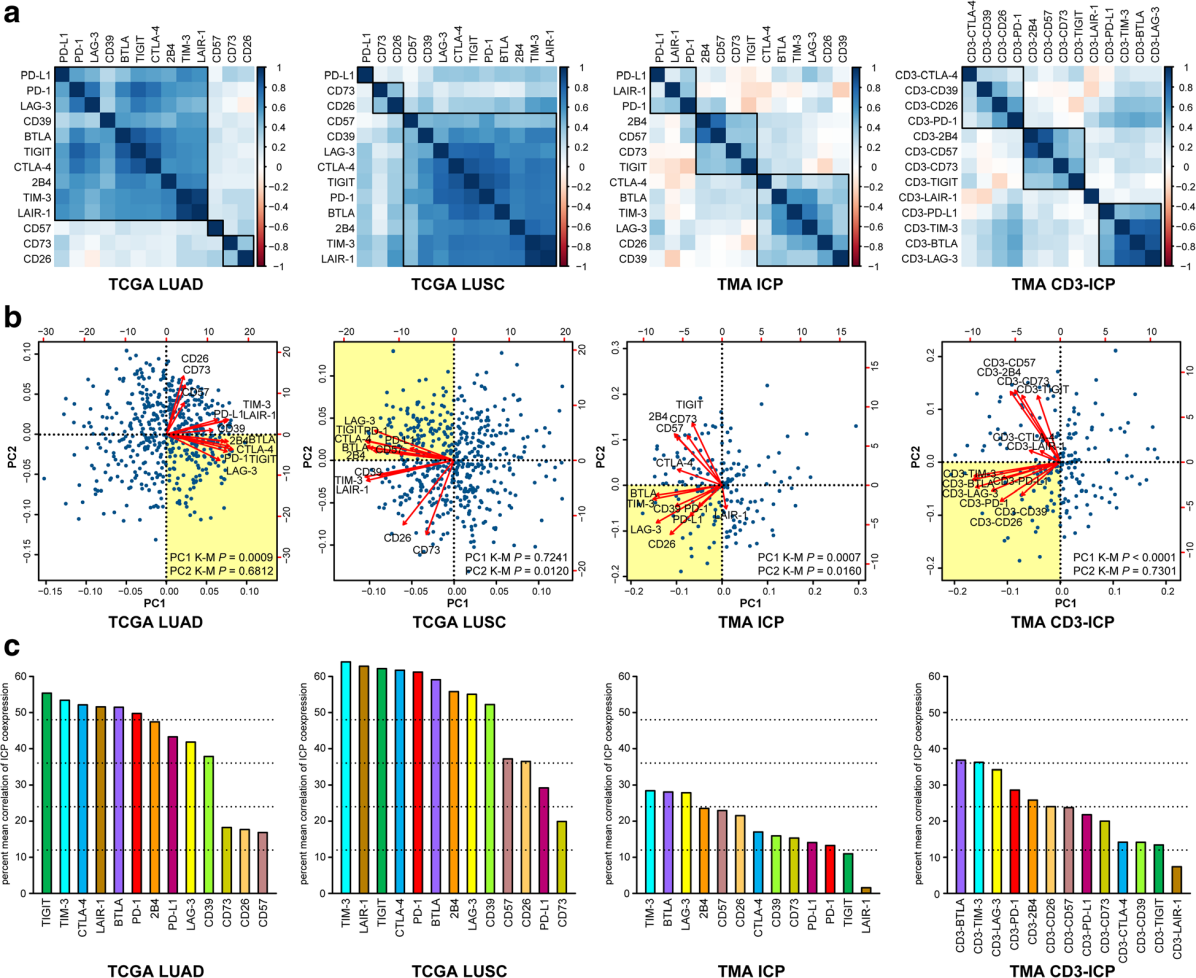
RNA and protein conserved ICP coexpression groups ranked for NSCLC patient stratification. **a**-**c** Graphs depicting R package generated correlation studies made between all ICPs from RNA and TMA datasets to reveal ICP coexpression dynamics stratifying patients. From left to right, RNA expression of ICPs from the TCGA LUAD (*n* = 504) and LUSC (*n* = 494) patient samples (left two graph columns), were compared to that of ICP and CD3-ICP expression from all TMA dataset patient (*n* = 73) samples (right two graph columns). **a** Correlograms demonstrating ICP coexpression clustering, where black boxes demarcate most highly correlating ICP. **b** PCA for visualization of multi-dimensional ICP coexpression, relative to distributions patient data (blue circles), where yellow shaded PC quadrants are occupied by ICP coexpressing groups having positive associations with OS, defined by Additional file 1: Figure S7. **c** Mean correlations of ICP coexpression demonstrate those most abundantly expressed relative to all other ICPs in NSCLC patients. Analyses were performed using alternative software (see Online Methods) by two independent operators. PC1, principal component 1; PC2, principal component 2

From the demonstration that specific combinations of ICPs could efficiently stratify patients, we performed correlation studies between all ICPs from RNA and TMA datasets to reveal ICP coexpression dynamics (Additional file 1: Table S13). Correlograms showed that for both RNA datasets, a majority of ICPs were highly correlated in expression (Fig. 6a), with the most highly correlating pairs being TIM-3 and LAIR-1, and CTLA-4 and TIGIT. TMA cohort correlograms reveal strongest associations between 2B4 and CD57, and BTLA, TIM-3 and LAG-3; this group conserved across all four datasets, and positively associating with OS.

PCA was deployed to better define synergizing ICPs across different MP-IF panels (Fig. 6b). Proportions of variance of principle components (PC), corresponding to combined expression of each ICP group, validated that the first PC (PC1), followed by the second PC (PC2), accounted for the greatest degrees of variance – representing groups having differential and unrelated expression dynamics (Additional file 1: Figure S7a). K-M was computed using high vs low PC group values (Additional file 1: Figure S7b). From the TMA dataset, a group of highly expressed ICP (low PC1) was significantly associated with OS (*P* = 7.3 × 10^− 4^). The relationship between PC1 and OS was increased using CD3-ICP values (*P* = 1.4 × 10^− 5^). PC2 values representing the second ICP cluster did not demonstrate as clear a relationship with survival. Altogether, this analysis revealed that the coexpressing ICP group BTLA^+^LAG-3^+^PD-1^+^PD-L1^+^ most efficiently stratified patients across all datasets (Fig. 6b and Additional file 1: Table S14). The TIGIT^+^CTLA-4^+^2B4^+^ group was maintained across RNA datasets, and the TIM-3^+^CD26^+^CD39^+^ group was maintained across protein datasets.

We performed correlation analyses to determine which ICPs were most highly coexpressed. For RNA datasets, ICP ranking was TIM-3-TIGIT-CTLA-4-LAIR-1-BTLA-PD-1 (Fig. 6c). For TMA protein-derived datasets, this was BTLA-TIM-3-LAG-3-PD-1. In our comparison of four cancers, CTLA-4-TIGIT-PD-1-TIM-3-BTLA- LAG-3 were among those most increased in expression and having the greatest association with OS (Additional file 1: Tables S7 and S8). Additional file 1: Figure S8 demonstrates detection of ICPs from whole-tumor RNA to protein on TMA CD3^+^ TILs, where augmented ICPs may be at the forefront of the anti-cancer response, making these the best CDx and ICP-blockade targets. To determine whether coexpression dynamics could be reflected by time to effect on OS, we examined K-M curves to identify ICPs having the earliest effect on OS. For both RNA and protein datasets, ICPs with the greatest impact on OS, either alone or in combination (Figs. 3, 5 and 6), were among those having the earliest impact on OS (Additional file 1: Figure S9). Taken together, these results revealed that key ICP groups have conserved coexpression from whole-tumor RNA to protein on TILs, where discrepancies may arise from ICP expression by other cells of the tumor microenvironment also captured by whole-tumor RNA datasets. The prevailing conserved ICP subgroup (BTLA/TIM-3/LAG-3/PD-1) was most highly coexpressed and had the largest impact on OS. It is not known whether these ICPs are the first accumulating, or those persisting longest on TILs, but these are surely robust targets for combination CDx.

### Redundant ICP-interacting proteins are linked with NSCLC patient survival

**Fig. 7:**
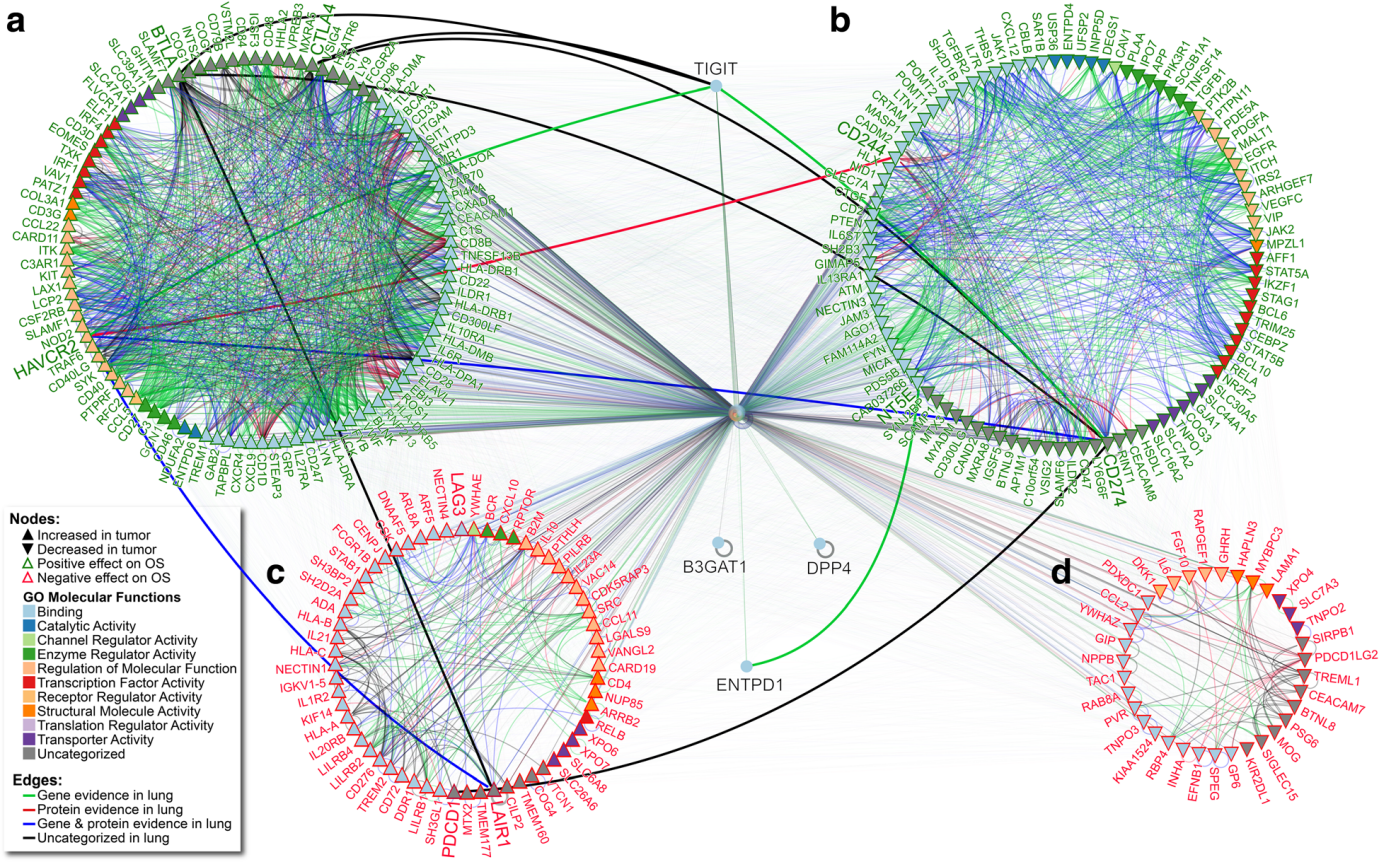
ICP-interacting proteins associated with NSCLC patient survival. Visualization of complete comprehensive and interactive ICP-ICP and ICP-proteins interaction mapping by NAViGaTOR. ICP interactors with (**a**) increased gene expression in tumors and positive association with OS, (**b**) decreased gene expression in tumors and positive association with OS, (**c**) increased gene expression in tumors and negative association with OS, and (**d**) decreased gene expression in tumors and negative association with OS

From the observation that ICPs positively associating with OS were increased in expression in tumor samples (Additional file 1: Table S8), we used the Integrated Interaction Database (IID) to identify 1750 key ICP-protein interactions from 40,555 possible interactions between all identified ICP-interacting proteins. Key ICP-interactors were refined for those that were 1) experimentally validated to interact with ICP, 2) redundantly interacting with more than one ICP, 3) associated with OS, and 4) had supporting evidence for their interactions in lung tissues (Additional file 1: Table S15). NAViGaTOR software was used to visualize all ICP-interactors, their characterized molecular functions, and supported interactions in lung tissues; demonstrating that 10 of the 13 signature ICP interacted with each other (Additional file 1: Figure S10, Table S16, and Additional file 4). Interaction networks were expanded to visualize defined groups from refined ICP-interactors (Fig. 7). The majority of ICP-interactors had a positive association with OS (64.6%); most of which also had increased gene expression in tumors (85.4%). The majority of ICPs in these two categories were also those ranking highest in interactions with other ICPs. Both increased in expression in tumors and associated with positive OS, BTLA and TIM-3 were observed to interact with a majority of these proteins (Fig. 7 and Additional file 1: Table S15). The pathDIP portal was used for comprehensive pathway enrichment analyses of ICP-ICP interactions and refined ICP-interactors lists (Fig. 7 and Additional files 3 and Additional file 4), and word-cloud analysis was used to compile the most significant ICP-interactors and associated pathways (Additional file 1: Figure S11). Together, these results demonstrate that most ICP-interactors are increased in expression and are associated to positive outcome, further suggesting that ICPs are positive prognostic NSCLC biomarkers.

## Discussion

ICPs were originally classified as exhaustion markers of functionally impaired T cells. Investigations of this reversible impairment have led to numerous clinical successes in cancer treatment. We were initially surprised that ICP expression on NSCLC TILs was positively associated with survival; a finding we confirmed using several additional cohorts spanning different solid cancers. When assessed in combinations, PD-1 and PD-L1 are positive prognostic markers of effector memory antigen-experienced CD8^+^ T cells [42]. ICP expression kinetics have been suggested to reflect CD8^+^ T cell differentiation kinetics rather than functional impairment [43], and as also suggested by our results, these are speculated to accumulate on TIL in an ordered fashion, led by PD-1, TIM-3, CTLA-4, LAG-3, and BTLA [44]. These represent robust CDx candidates because their prognostic/stratifying effects are also visible using whole-tumor RNA datasets. Another recent study by the Zippelius group is an additional demonstration of the rethinking of the meaning of T cell exhaustion/dysfunction in NSCLC, demonstrating that NSCLC TIL populations coexpressing several ICPs are highly clonal with a predominance of TCRs resulting from their antigen-driven expansion, that these secrete high levels of chemokines recruiting B cell and CD4+ helper cells into tumors, but most importantly, that this population is a strong predictor of robust responses to immunotherapy and overall survival [45].

We identify BTLA as the most reproducible prognostic biomarker spanning all cohorts investigated, as it: 1) predicted positive outcome from the TMA; 2) predicted positive outcome from whole-tumor RNA; 3) was most coexpressed with other ICPs across all datasets; 4) had earliest effects on OS; 5) had increased expression in tumors; 6) interacted with a majority of other ICPs and other proteins; and 7) was almost exclusively expressed by TILs. Responders to adoptive cell transfer (ACT) have increased proportions of CD8^+^BTLA^+^TIM-3^+^ TIL infusion products [46], and BTLA is speculated to be the final checkpoint towards differentiation into effector T cells [47]. Accordingly, BTLA was the only ICP decreased from stimulation ahead of transfusion of autologous cultures used for successful NSCLC ACT [48, 49]. BTLA may be an ideal target for ICP-blockade, because it is restricted to lymphoid tissues, and its inhibition restores TCR signaling [50]. BTLA protects TILs from apoptosis [51], and with T cell longevity estimated at over a decade [52], balanced BTLA expression may make the difference between antigen-experience and death.

Even using large biospecimens, the heterogeneity of the tumor microenvironment is the biggest challenge to finding prognostic and predictive biomarkers. We have thus developed a method for stratifying patients from limited biospecimens unsuitable for standard IM. Our restriction of analysis to immune-dense regions overcomes both size and heterogeneity of biospecimens, identifying several IIC and ICP combinations stratifying NSCLC patients. This fully-automatable combination CDx platform represents an optimal salvage method for profiling TILs from baseline biopsies ahead of personalized ICP-blockade therapies. The BTLA, TIM-3, LAG-3 and PD-1 combination on TILs was increased in expression and offered the best survival advantage. These ICPs were among those having: 1) highest correlation with any other ICP on CD3^+^ TILs, 2) positive association with OS at both RNA and protein levels, 3) the earliest effects on K-M curves, 4) equal impact on OS from the alternative method of analysis, and 5) decreased expression at advanced stages. These ICP may be among the first, or most persistently expressed by TILs gaining antigenic experience, as suggested by their strong correlation with TIL-Bs. This ICP subgroup represents the best CDx combination for stratifying patients using small biospecimens.

This work was in part performed to address the issues plaguing PD-L1 as CDx. Demonstrations of PD-L1 contribution to disease is challenging because it is easily inducible or constitutively expressed by many cell types. We observed that PD-L1 only stratified patients when co-labeling with CD8 or TIM-3. Likewise, despite initially described as a poor prognostic factor, PD-L1 association with TILs is linked to better outcomes in diverse cancer types [53, 54], and its expression on TILs predicts response to α-PD-L1 [55, 56]. Our finding that CD3-PD-L1 association with OS was affected by the alternative method of analysis confirms variability of PD-L1 expression on TIL within individual biopsies. Conversely, associations of CD8-PD-L1 and TIM-3-PD-L1 with OS was unaffected, substantiating little variability in their co-occurrences. Success of PD-L1 as CDx may thus not come down to the choice of clone, but rather from its profiling in combinations providing adequate ‘immune contexture’. Like PD-L1, we find that numerous ICPs and IICs better stratify patients when profiled in combination.

Despite ICP being excellent targets for immunotherapies, they are also crucial for T cell survival. Our study does not aim to invalidate reports of ICPs as inhibitory receptors: Indeed, certain solitary ICP from whole-tumor RNA-datasets are associated with negative outcomes. Nonetheless, evidence that the majority of redundant ICP-interactors positively associate with outcomes implies ICPs have numerous important functional roles for T cells (Additional file 1: Table S17). In relation to our findings that TIL-Bs correlate with ICP coexpression and inversely correlate with metastasis, ADC clonal neoantigen-enriched tumors are significantly associated to OS, have increased ICP expression, and are more sensitive to blockade therapies [57]. Specific ICP combinations may accumulate on TILs actively becoming educated against clonal neoantigens, and may protect TILs from apoptosis by slowing metabolism and differentiation kinetics. Robust MP-IF ICP CDx may identify TILs primed for tumor elimination, and the best targets for personalized immunotherapies. MP-IF ICP CDx may be also used to monitor ICP repertoires of tumor-reactive TIL expansion products for ACT. MP-IF ICP CDx created according to ICP ranking can anticipate additional ICPs arising during immunotherapies, and improve response rates to mono- and combo-ICP-blockade towards their permanent adoption by mainstream oncology.

## Conclusions

In this hypothesis-generating study, deepening our understanding of immune-checkpoint biology, comprehensive protein-protein interaction and pathway mapping revealed that redundant immune-checkpoint interactors associate with positive outcomes, providing new avenues for deciphering the effects of immunotherapies. We find combinations that efficiently stratify patients, and validate prognostic ICP-signatures on additional cohorts. We profile ICP coexpression dynamics and ICP linkage to clinical parameters and IIC subsets, map ICP-interactors and associated pathways, and define the most prognostic combinations that can guide blockade therapies using baseline biospecimens of all sizes.

## Additional files

Additional file 1: **Figure S1.** Methods, antibodies and validation that IIC-density does not correlate with patient clinicopathological characteristics. **Figure S2.** CD45 distribution on TMA cores and K-M plots of immune cells and Ki-67. **Figure S3.** Five-color panels demonstrate which ICP subsets preferentially label TIL. **Figure S4.** Effect of applied treatments on OS of TMA patient cohort. **Figure S5.** Validation of effect of ICP signature on additional NSCLC cohorts and cancers. **Figure S6.** MP-IF ICP combination panels stratifying NSCLC patients. **Figure S7.** Kaplan-Meier survival analysis of principle components positively associated with OS. **Figure S8.** ICP coexpression ranking demonstrates ICP subset. **Figure S9.** Timing of effects of ICP expression on Kaplan-Meier survival curves. **Figure S10.** Compressed view of refined ICP-interactors presented in Fig. 7. **Figure S11.** Word-cloud analysis of top ICP interactors and associated pathways. **Table S1.** Clinicopathologic characteristics of TMA cohort. **Table S2.** Correlations between clinicopathological characteristics of TMA cohort. **Table S3.** Antibodies used in the study. **Table S4.** Correlations of TMA ICP MFI with immunogenicity and clinical characteristics. **Table S5.** Correlations of TMA ICP counts with immunogenicity and clinical characteristics. **Table S6.** Primary validation of positive association of ICP expression with OS. **Table S7.** Secondary validation of positive association of ICP expression with OS. **Table S8.** ICP RNA expression in normal vs. cancer tissues. **Table S9.** Validation of increased positive association with OS by ICP-TIL combination. **Table S10.** Validation of effect of IIC expression on OS. **Table S11.** Chromosomal locations of profiled ICP. **Table S12.** Association of TMA ICP combinations from MP-IF panels with OS. **Table S13.** Figure 5a correlogram common ICP groupings. **Table S14.** Figure S7 PC1 and PC2 groups positively associated with OS. **Table S15.** ICP- interactors having effects on K-M and modulated in their expression. **Table S16.** ICP-ICP interactors from IID. **Table S17.** Positive T cell functions of selected NSCLC patient stratifying ICPs. (ZIP 10706 kb)

Additional file 2: ICP and annotations on pathways profiles. (XLSX 146 kb)

Additional file 3: Refined ICP-interactor annotations on pathways profiles. (XLSX 5574 kb)

Additional file 4: Interactive NAViGaTOR .n3e file of ICP-interactors and pathways. (N3E 958 kb)

## Abbreviations

ACT: Adoptive cell transfer
ADC: Adenocarcinoma
APC: Antigen presenting cells
CD3-ICP: ICP expressed on CD3^+^ TIL
CDx: Companion diagnostics
CTLA-4: Cytotoxic T lymphocyte-associated antigen 4
EGA: European Genome-phenome Archive
GEO: Gene Expression Omnibus
GZMB: Granzyme B
HEV: High endothelial venules
HLA-DR: Human leukocyte antigen-DR
ICP: Immune checkpoint
IF: Immunofluorescence
IFN-γ: Interferon-gamma
IHC: Immunohistochemistry
IIC: Infiltrating immune cells
IID: Integrated Interaction Database
IM: Immunoscore
K-M: Kaplan-Meier survival analysis
LUAD: Lung adenocarcinoma
LUSC: Lung squamous cell carcinoma
MFI: Mean fluorescence intensity
MP-IF: Multiplex immunofluorescence
NAViGaTOR: Network Analysis, Visualization and Graphing, TORonto
NK cells: Natural killer cells
NSCLC: Non-small cell lung carcinoma
OS: Overall survival
pathDIP: Pathway Data Integration Portal
PD-1: Programmed death-1
PD-L1 and PD-L2: Programmed death-1 ligands 1 and 2
PNAd: Peripheral node addressin
SCC: Squamous-cell carcinoma
TAM: Tumor-associated macrophages
TCGA: The Cancer Genome Atlas
TCR: T cell receptor
TIL: Tumor infiltrating lymphocytes
TMA: Tissue microarray
TNM: Tumor, node, metastases
