# Supplementary material for: Immune-enrichment of non-small cell lung cancer baseline biopsies for multiplex profiling define prognostic immune checkpoint combinations for patient stratification
Source: J Immunother Cancer. 2019 Mar 28;7:86. doi: 10.1186/s40425-019-0544-x (PMC6437930; doi:10.1186/s40425-019-0544-x)
Supplement: Supplementary file 1 — Figure S1. Methods, antibodies and validation that IIC-density does not correlate with patient clinicopathological characteristics. Figure S2. CD45 distribution on TMA cores and K-M plots of immune cells and Ki-67. Figure S3. Five-color panels demonstrate which ICP subsets preferentially label TIL. Figure S4. Effect of applied treatments on OS of TMA patient cohort. Figure S5. Validation of effect of ICP signature on additional NSCLC cohorts and cancers. Figure S6. MP-IF ICP combination panels stratifying NSCLC patients. Figure S7. Kaplan-Meier survival analysis of principle components positively associated with OS. Figure S8. ICP coexpression ranking demonstrates ICP subset. Figure S9. Timing of effects of ICP expression on Kaplan-Meier survival curves. Figure S10. Compressed view of refined ICP-interactors presented in Fig. 7. Figure S11. Word-cloud analysis of top ICP interactors and associated pathways. Table S1. Clinicopathologic characteristics of TMA cohort. Table S2. Correlations between clinicopathological characteristics of TMA cohort. Table S3. Antibodies used in the study. Table S4. Correlations of TMA ICP MFI with immunogenicity and clinical characteristics. Table S5. Correlations of TMA ICP counts with immunogenicity and clinical characteristics. Table S6. Primary validation of positive association of ICP expression with OS. Table S7. Secondary validation of positive association of ICP expression with OS. Table S8. ICP RNA expression in normal vs. cancer tissues. Table S9. Validation of increased positive association with OS by ICP-TIL combination. Table S10. Validation of effect of IIC expression on OS. Table S11. Chromosomal locations of profiled ICP. Table S12. Association of TMA ICP combinations from MP-IF panels with OS. Table S13. Figure 5a correlogram common ICP groupings. Table S14. Figure S7 PC1 and PC2 groups positively associated with OS. Table S15. ICP- interactors having effects on K-M and modulated in their expression. Table S16. ICP-ICP [file 40425_2019_544_MOESM1_ESM.zip › Supplementary_MONETTE_JITC R1.docx]

Supplementary Figures and Tables for:

Immune-enrichment of non-small cell lung cancer baseline biopsies for multiplex profiling define prognostic immune checkpoint combinations for patient stratification

This file includes

Supplementary Figures:

1: Methods, antibodies and validation that IIC-density does not correlate with patient clinicopathological characteristics.

2: CD45 distribution on TMA cores and K-M plots of immune cells and Ki-67.

3: Five-color panels demonstrate which ICP subsets preferentially label TIL.

4: Effect of applied treatments on OS of TMA patient cohort.

5: Validation of effect of ICP signature on additional NSCLC cohorts and cancers.

6: MP-IF ICP combination panels stratifying NSCLC patients.

7: Kaplan-Meier survival analysis of principle components positively associated with OS.

8: ICP coexpression ranking demonstrates ICP subset.

9: Timing of effects of ICP expression on Kaplan-Meier survival curves.

10: Compressed view of refined ICP-interactors presented in Figure 7.

11: Word-cloud analysis of top ICP interactors and associated pathways.

Supplementary Tables:

1. Clinicopathologic characteristics of TMA cohort.

2. Correlations between clinicopathological characteristics of TMA cohort.

3. Antibodies used in the study.

4. Correlations of TMA ICP MFI with immunogenicity and clinical characteristics.

5. Correlations of TMA IIC and ICP counts with immunogenicity and clinical characteristics.

6. Primary validation of positive association of ICP expression with OS.

7. Secondary validation of positive association of ICP expression with OS.

8. ICP RNA expression in normal vs. cancer tissues.

9. Validation of increased positive association with OS by ICP-TIL combination.

10. Validation of effect of IIC expression on OS.

11. Chromosomal locations of profiled ICP.

12. Association of TMA ICP combinations from MP-IF panels with OS.

13. Figure 5A correlogram common ICP groupings.

14: Fig S7 PC1 and PC2 groups positively associated with OS.

15. ICP- interactors having effects on K-M and modulated in their expression.

16. ICP-ICP interactors from IID.

17. Positive T cell functions of selected NSCLC patient stratifying ICPs.

**Supplementary Figure 1: Methods, antibodies and validation that IIC-density does not correlate with patient clinicopathological characteristics.**


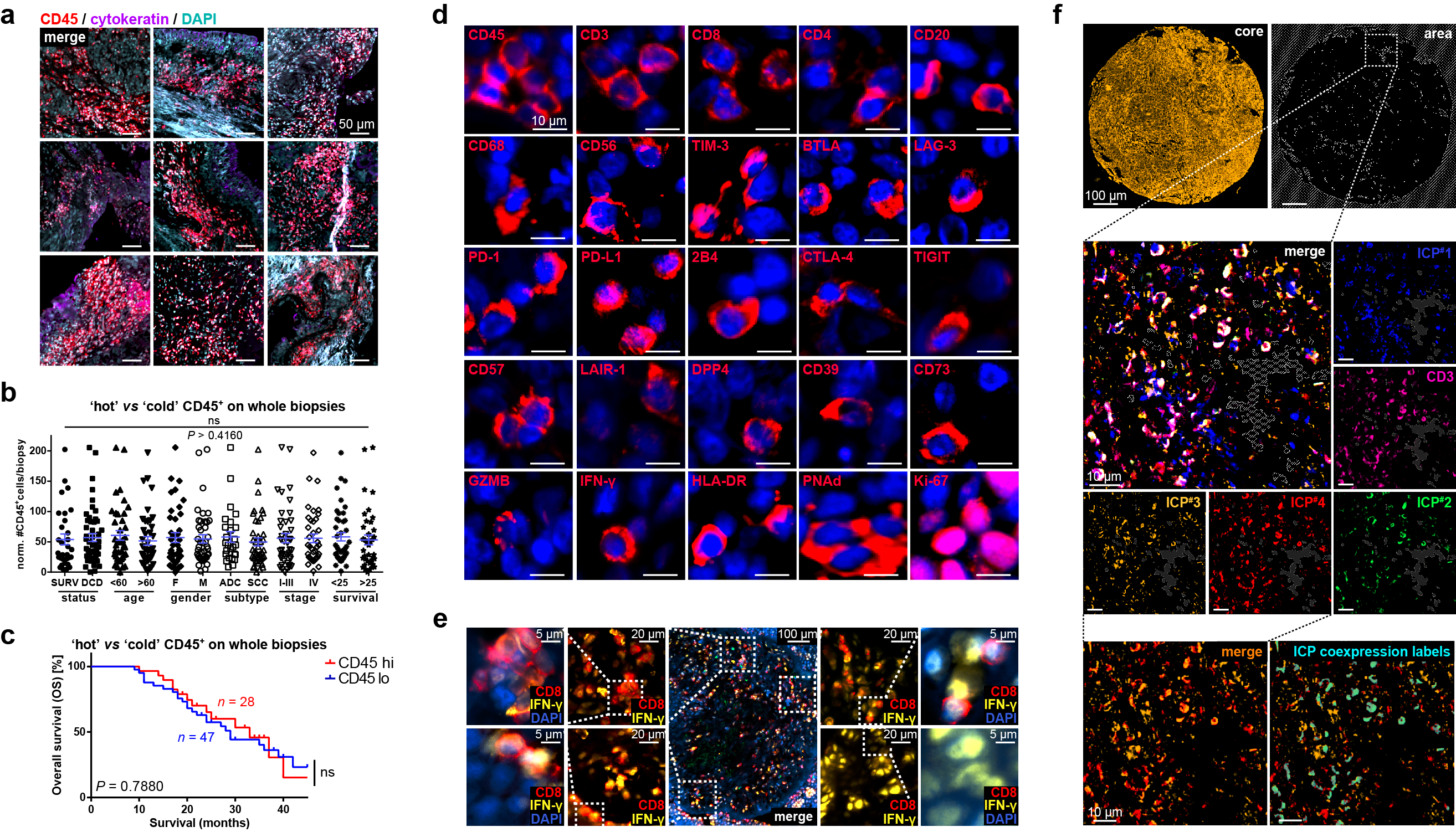


(**a**) Whole biopsy sections were immunostained for IF using α-CD45, α-cytokeratin 8/18 (EP17), and DAPI (*n* = 9). (**b**) Graph demonstrating no association between immune density of biopsies (high, “hot”; low, “cold”; *P* > 0.4160; *n* = 81) and clinicopathological characteristics (SURV, surviving (*n* = 31); DCD, deceased (*n* = 50); <60, under 60 years of age (*n* = 39); >60, over 60 years of age (*n* = 42); F, female (*n* = 39); M, male (*n* = 42); ADC, Adenocarcinoma (*n* = 26), SCC, Squamous cell carcinoma (*n* = 42); stage I-III (*n* = 42); stage IV (*n* = 39); <25, under 25 months survival (*n* = 43); >25, over 25 months survival (*n* = 38); Student's t test; error bars represent mean ± s.e.m.. (**c**) K-M of OS relative to IIC-density of biopsies. (**d**) Commercial antibodies validated on formalin-fixed paraffin-embedded (FFPE) tissues, are listed in Supplementary Table 3. These were individually tested for their antigen-labeling specificities on large FFPE tumor slide sections. Secondary Alexa-Fluor 594 antibodies were used to detect primary antibodies. With the exception of nuclear staining Ki-67, punctate cytoplasmic staining GZMB, and IFN-γ (**e**), all antibodies tested were found to predominantly stain the cytoplasm of lymphocytes surrounding nuclei stained by DAPI (blue). Of the three IFN-γ antibodies tested, ab25101, shown here, provided specific staining and association with OS. (**f**) Example of algorithms created to define core areas and create ICP coexpression labels for quantification. μm, micron; norm., normalized; *P*, log-rank test; ns, not significant; *n*, number of patients; CD45 hi, high CD45 density; CD45 lo, low CD45 density.**Supplementary Figure 2: CD45 distribution on TMA cores and K-M plots of immune cells and Ki-67.**


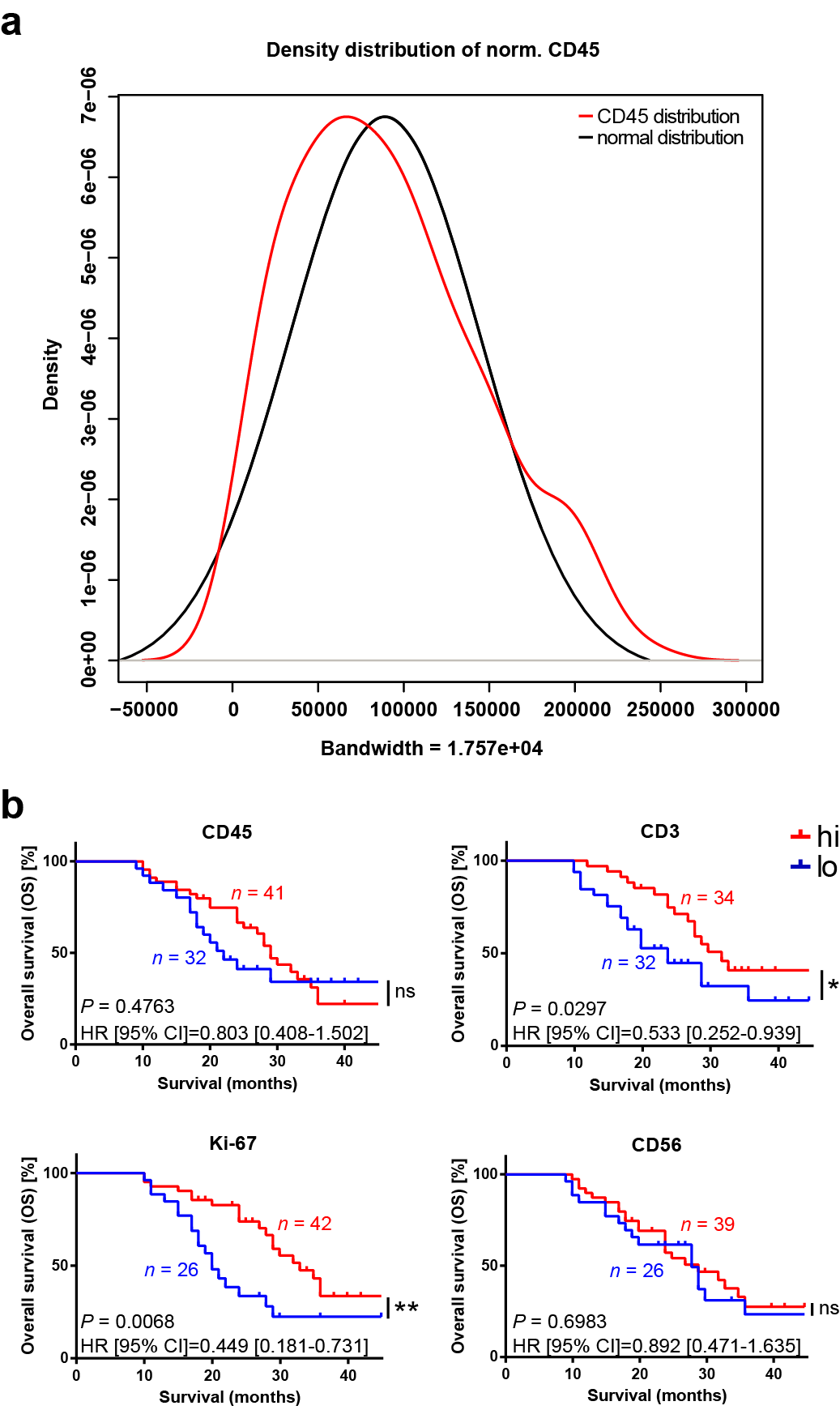


**(a)** The distribution of core-area normalized (norm.) CD45 on TMA cores was compared to the normal distribution. **(b)** K-M association with OS curves by pan IIC marker CD45, T cell marker CD3, proliferation marker Ki-67, and NK cell marker CD56. The number of patients (*n*) for each group is given on K-M curves. *P*, Log-rank test; ns, not significant; * *P* < 0.05; ** *P* < 0.01; HR, hazard ratio (Log-rank); CI, confidence interval of ratio.**Supplementary Figure 3: Five-color panels demonstrate which ICP subsets preferentially label TIL.**


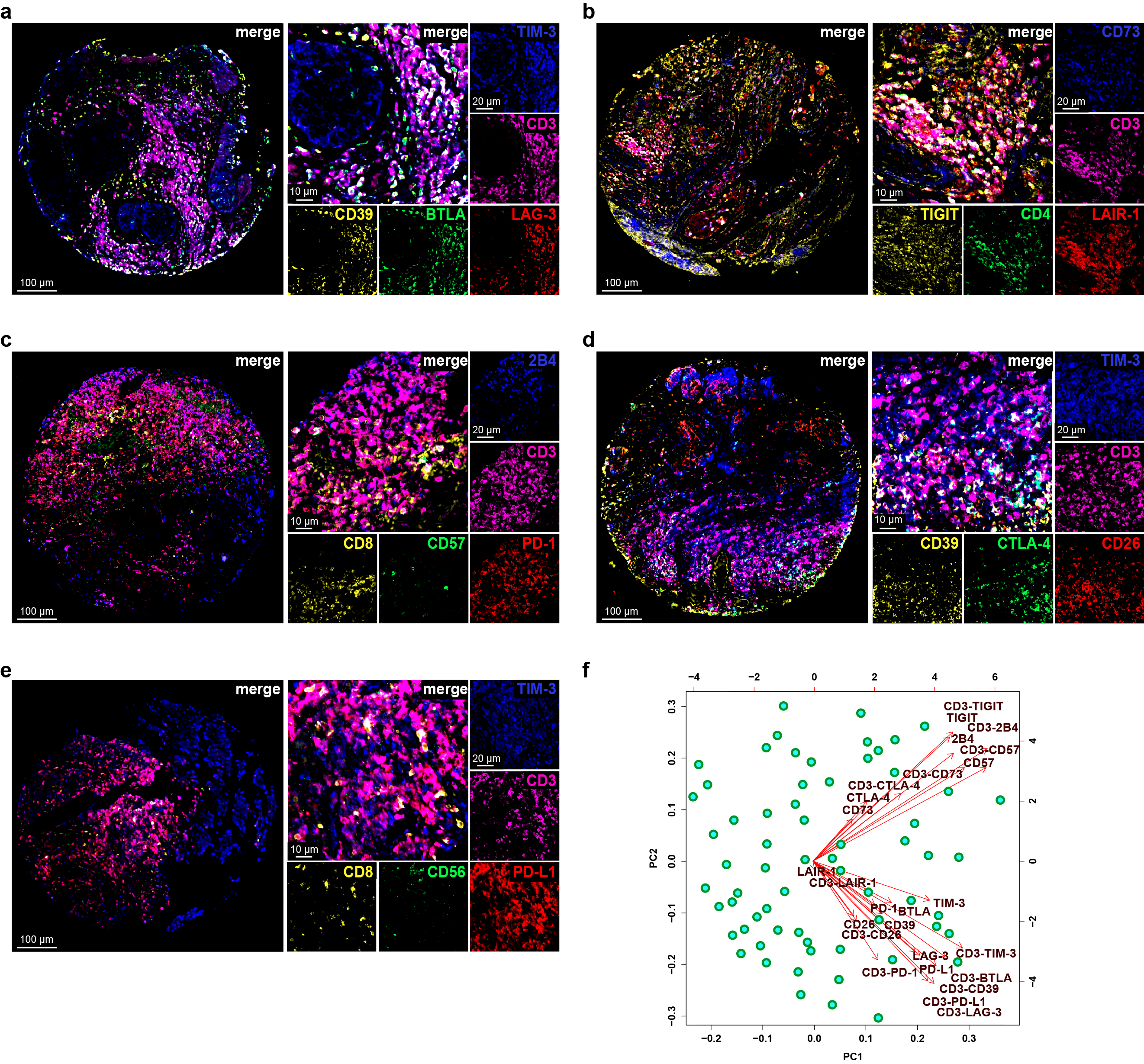


**(a-e)** Representative TMA cores demonstrate that CD39, BTLA, LAG-3, CD57, PD-1, 2B4, CTLA-4 and CD26 preferentially label TIL, whereas TIM-3, PD-L1, CD73, TIGIT and LAIR-1 label numerous cells of the tumor microenvironment. μm, micron; merge, merge of all IF channels. **(f)** PCA of ICP and CD3-ICP clustering according to their expression on patients (green dots).**Supplementary Figure 4: Effect of applied treatments on OS of TMA patient cohort.**
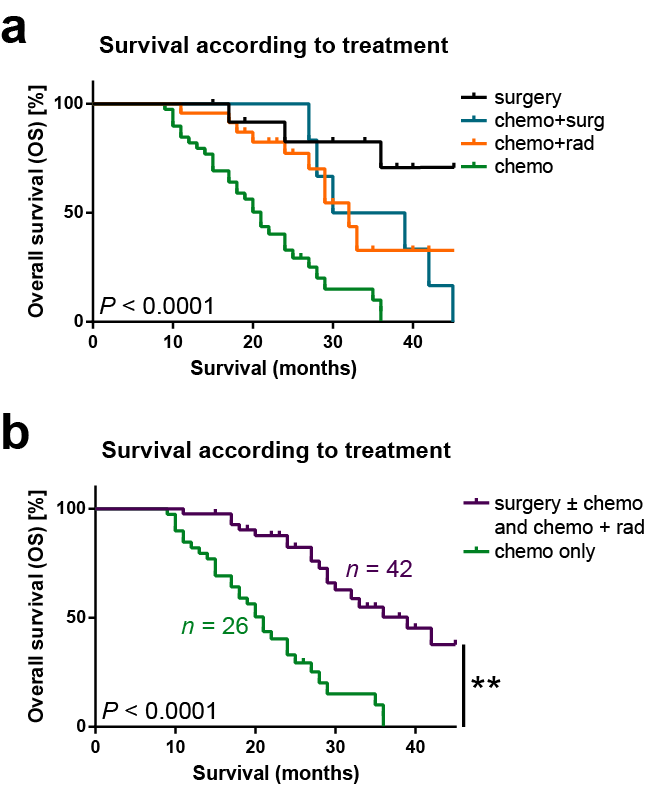


**(a)** K-M analysis performed according to four individual treatments applied, or **(b)** according to chemotherapy only relative to all other treatments and combinations of thereof. P, Log-rank test, chemo, platinum-based chemotherapy, rad, radiation treatment.**Supplementary Figure 5: Validation of effect of ICP signature on additional NSCLC cohorts and cancers.**


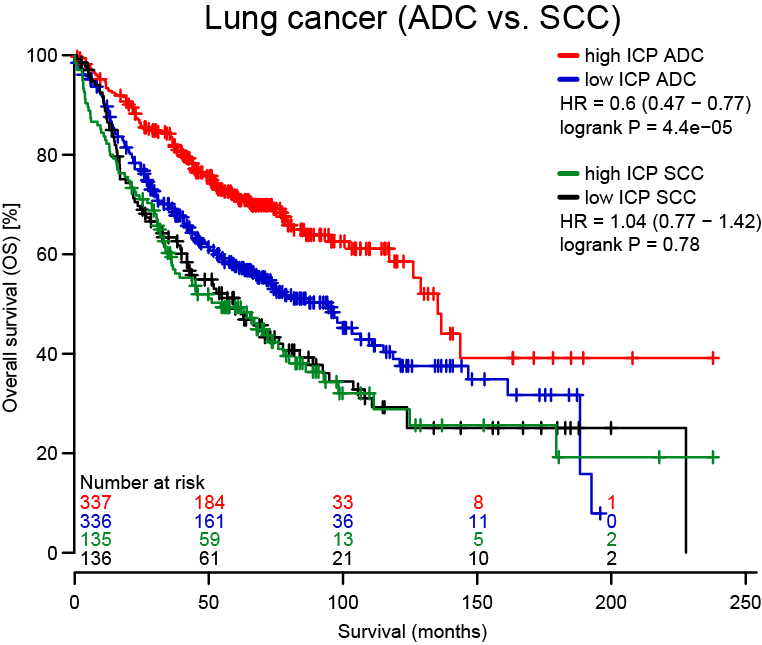


GEO, EGA and TCGA microarray validation cohorts were used to perform K-M analysis for on NSCLC ADC (*n* = 673) *vs.* SCC subtypes (*n* = 271). HR, hazard ratio (Log-rank) with 95 percent confidence interval.**Supplementary Figure 6: MP-IF ICP combination panels stratifying NSCLC patients.**
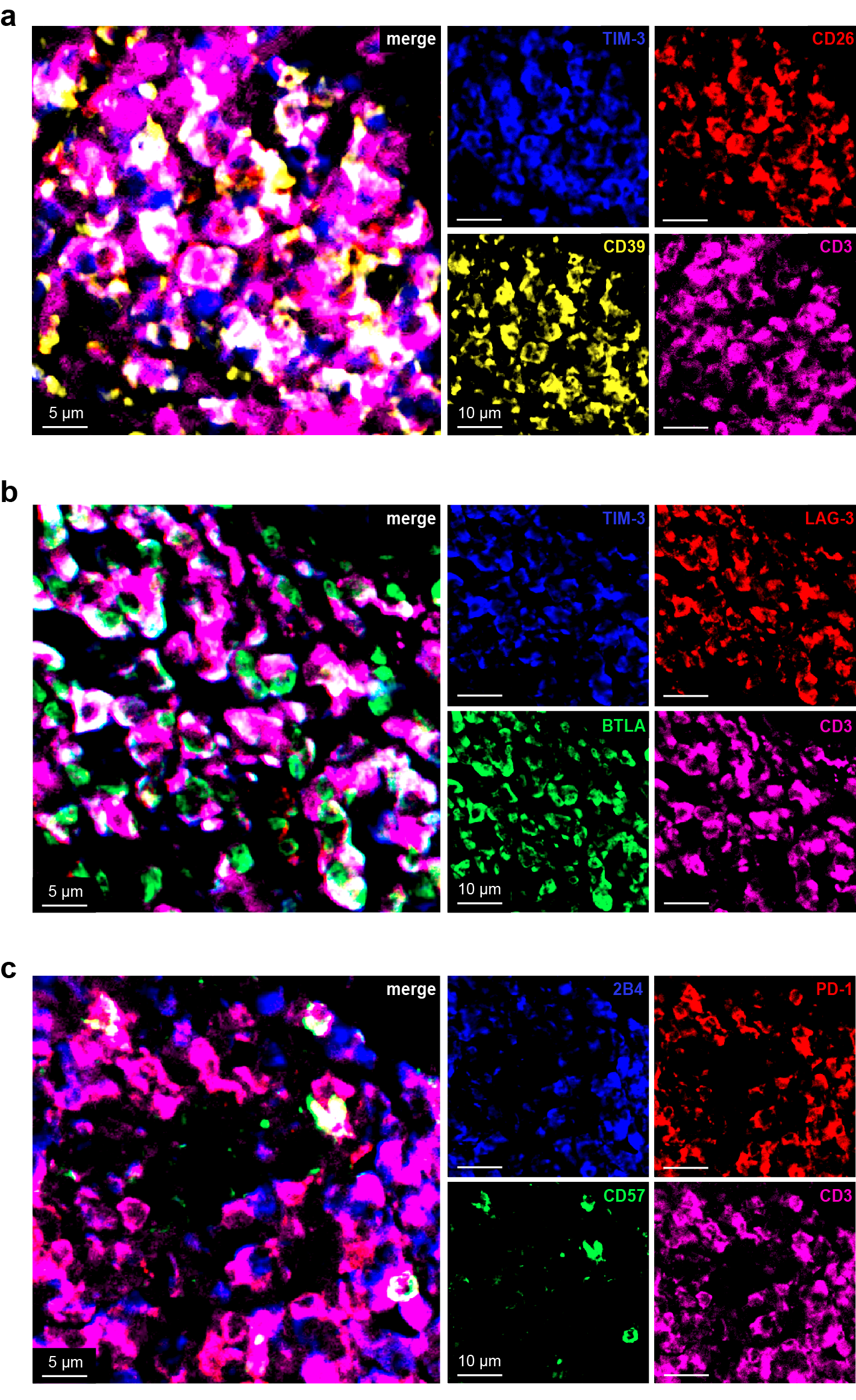


Accompanying representative images of CD3-ICP colocalization-dense core areas for Figure 5 (**a**) (TIM-3, CD26 and CD39), (**b**) (TIM-3, BTLA and LAG-3), and (**c**) (2B4, PD-1, and CD57). μm micron; merge, merge of all IF channels.

**Supplementary Figure 7: Kaplan-Meier survival analysis of principle components positively associated with OS.**


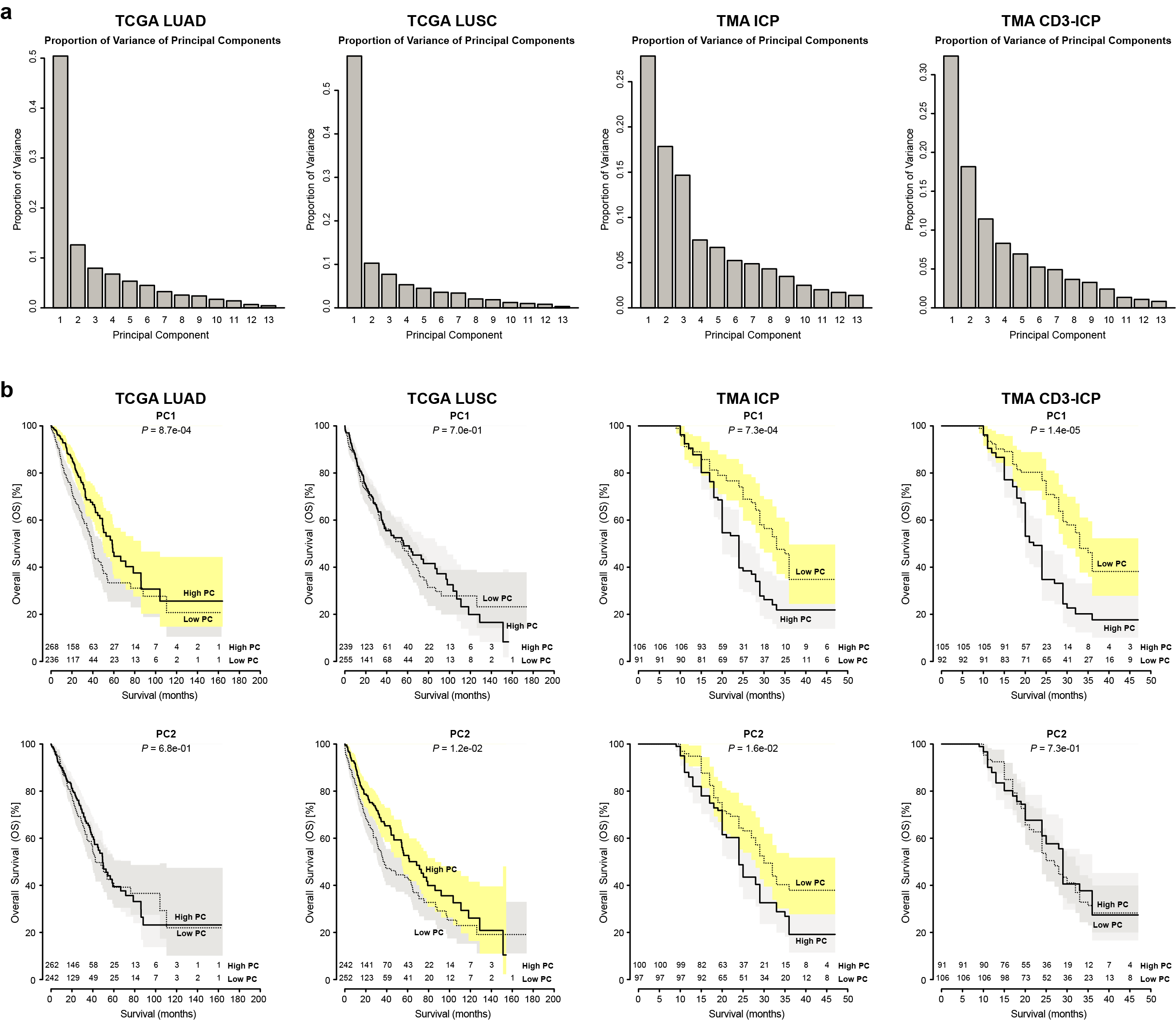


From left to right, ICP RNA expression data from the TCGA LUAD and LUSC datasets (left), and TMA ICP and CD3-ICP datasets (right), were used to define key ICP groups not formerly observed by MP-IF panels. **(a)** Proportions of variance of PC were examined to verify that PC1 and PC2 had the greatest variance relative to all other PC. From the TCGA RNA datasets, the ICP from patient samples accounting for the dominant PC1 variation vary in the same direction (BTLA, TIM3, LAG3, PD1, PDL1, CTLA4, CD39, TIGIT, LAIR1, CD244). Many are common to the ICP from patient samples accounting for the PC1 variation of for the TMA datasets (BTLA, TIM3, LAG3, PD1, PDL1, CTLA, CD39, CD26). A second group of ICP from patient samples (TIGIT, CD244, CD73, CD57) account for PC2 variation of TMA dataset. (**b**) PC1 and PC2 were used to assess the effects of coexpressing gene groups in their given quadrants on OS using K-M from **Fig. 1G**, where confidence intervals of PC having significance on OS from K-M are shaded in yellow as are quadrants presented in Fig. 5. High and Low PC on K-M curves are representative of the location of the gene groups found in Fig. 5 relative to PC1 and PC2 axes. Rows of numbers within graphs represent numbers of subjects at risk.**Supplementary Figure 8: ICP coexpression ranking demonstrates ICP subset.**
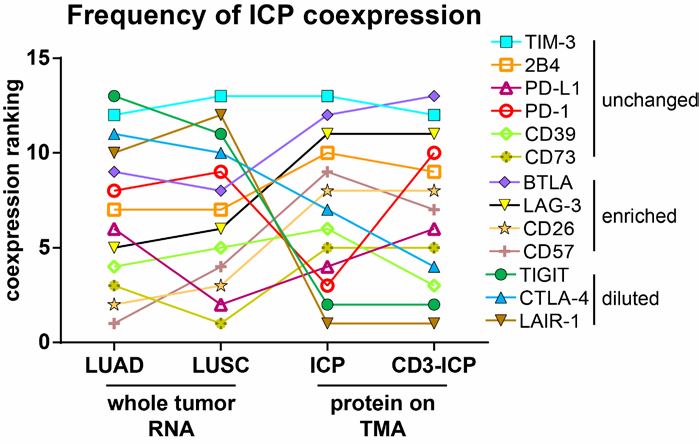


From left to right, ICP RNA expression data from the TCGA LUAD and LUSC datasets (left), and TMA ICP and CD3-ICP (right) datasets were used to determine which ICP may be most important to CD3^+^ TIL. Coexpression ranking on left axis reflects order of ICP observed from percent mean correlation of ICP coexpression from Figure 6c. ICP names are organized on right axis according to whether their ranking stays the same, is enriched, or is diluted as we move from RNA (left) to protein on CD3^+^ TIL (right) of x-axis.

**Supplementary Figure 9: Timing of effects of ICP expression on Kaplan-Meier survival curves.**

**
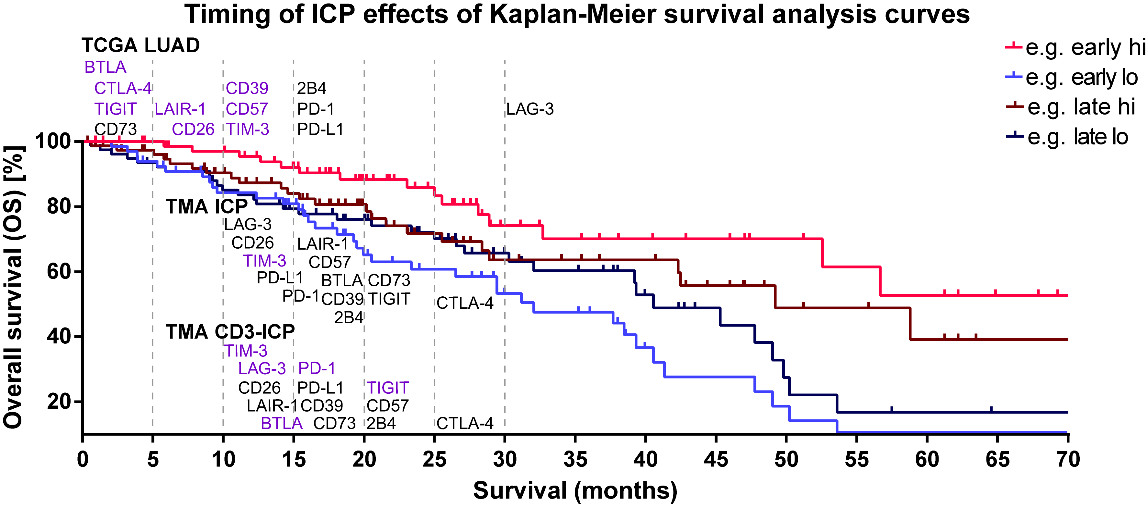
**

K-M curves for each ICP from the TCGA LUAD RNA dataset and TMA ICP and CD3-ICP datasets were examined to determine starting time point for separation of high and low ICP expression curves relative to patient survival times. Examples of ICP effects acting early “e.g. early hi or lo” (hi *vs* lo, high *vs* low) (from LUAD dataset BTLA; bright red and blue), or acting late “e.g. late hi or lo” (from LUAD dataset LAG-3; brown and dark blue) are given as visual examples of separating curves. Genes having a positive effect on OS are colored in purple.

**Supplementary Figure 10: Compressed view of refined ICP-interactors presented in Figure 7.**


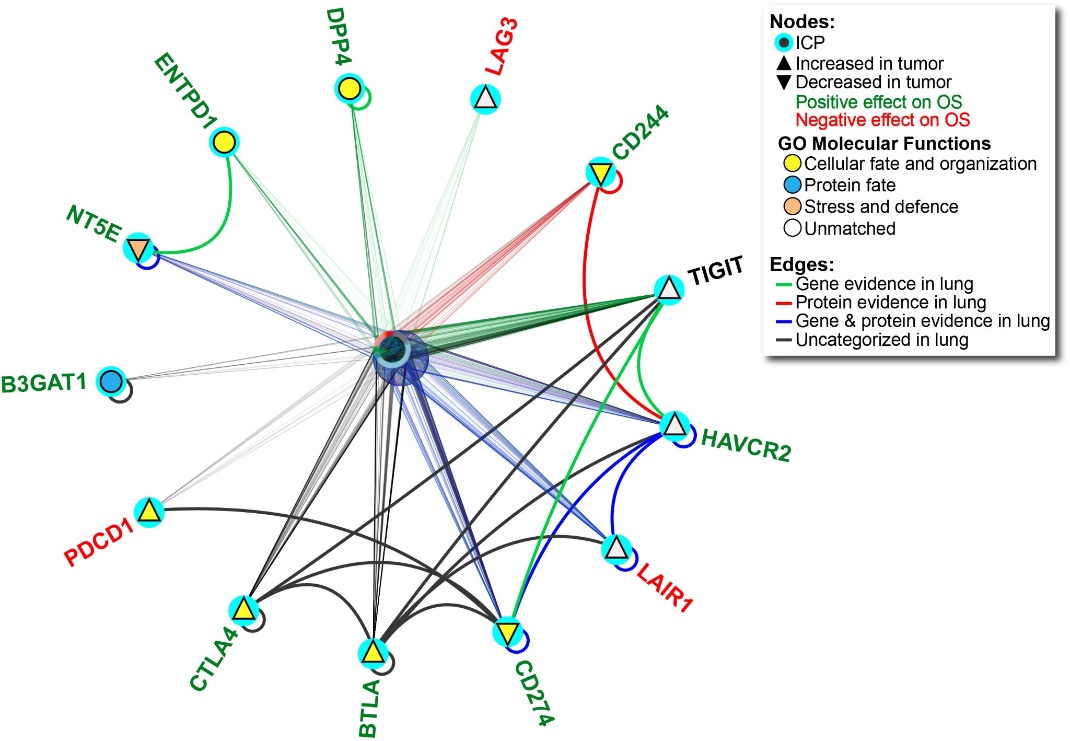


NAViGaTOR software highlights physical protein-protein interactions for ICP downloaded from IID, along with other lung proteins that are both modulated in expression and associated with NSCLC OS.

**Supplementary Figure 11: Word-cloud analysis of top ICP interactors and associated pathways.**


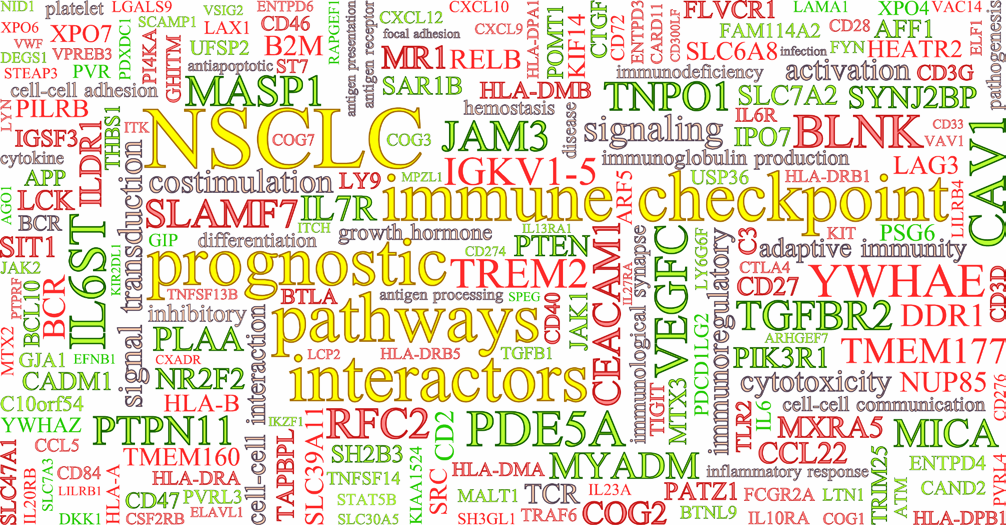


The most significant ICP-interactors from refined IID generated lists, and most significant associated pathways generated by pathDIP, were used to perform word-cloud analysis. Word sizes correspond to the frequency of pathway occurrence, and significance of gene expression modulation and impact on OS of NSCLC patients. Red colored genes are increased in expression in tumors, whereas green colored genes are decreased in tumors. Dark red and green outlines correspond to genes associated with positive OS.**Supplementary Tables:**

**Supplementary Table 1. Clinicopathologic characteristics of TMA cohort.**

| **Number of patients** | 81 patients | |
| --- | --- | --- |
| **Age** | Median 60 years | |
| **Overall survival** | Median 22 months | |
| **Parameters** | ***n*** | **%** |
| **Gender** |  |  |
| Male | 42 | 51.9 |
| Female | 39 | 48.1 |
| **Smoking history** |  |  |
| No | 31 | 38.3 |
| Yes | 50 | 61.7 |
| **T-stage** (primary tumor) |  |  |
| T1 | 0 | 0.0 |
| T2 | 21 | 25.9 |
| T3 | 10 | 12.3 |
| T4 | 50 | 61.7 |
| **N-stage** (lymph nodes) |  |  |
| N0 | 24 | 29.6 |
| N1 | 12 | 14.8 |
| N2 | 28 | 34.6 |
| N3 | 16 | 19.8 |
| Nx | 1 | 1.2 |
| **M-stage** (metastasis) |  |  |
| M0 | 35 | 43.2 |
| M1 | 41 | 50.6 |
| Mx | 5 | 6.2 |
| **UICC-stage** ǂ |  |  |
| I | 3 | 3.7 |
| II | 13 | 16.0 |
| III | 26 | 32.1 |
| IV | 39 | 48.1 |
| **Histological subtype** |  |  |
| Adenocarcinoma | 26 | 32.1 |
| Squamous cell carcinoma | 42 | 51.9 |
| NSCLC not specified | 13 | 16.0 |
| **Treatment** |  |  |
| surgery and chemotherapy | 6 | 7.4 |
| surgery | 13 | 16.0 |
| chemo and radiotherapy | 23 | 28.4 |
| chemotherapy | 39 | 48.1 |
|  | |  |

ǂ Union Internationale Contre le Cancer

**Supplementary Table 2. Correlations between clinicopathological characteristics of TMA cohort.**

| **Parameter** | **p-value*** | **r value** |
| --- | --- | --- |
| **Gender** (female *vs* male) |  |  |
| stage | 0.0215 | -0.2274 |
| survival time (months) | 0.0003 | 0.3526 |
| **Smoking history** (yes *vs* no) |  |  |
| UICC-stage | 0.0008 | 0.3278 |
| T-stage | 0.0334 | 0.2108 |
| M-stage | 0.0032 | 0.2965 |
| **T-stage** (T2-3 *vs* T4) |  |  |
| survival time (months) | 0.0003 | -0.0003 |
| survival event (DCD vs SURV) | 0.0020 | 0.2266 |
| M-stage | <0.0001 | 0.5392 |
| **M-stage** (pos *vs* neg for metastasis) |  |  |
| T-stage | <0.0001 | 0.5392 |
| **Age** (<60 *vs* >60) |  |  |
| subtype (ADC vs SCC) | 0.0007 | -0.3651 |
| survival event (DCD vs SURV) | 0.0077 | -0.2627 |
| **Survival event** (DCD *vs* SURV) |  |  |
| T-stage | 0.0220 | 0.2266 |
| M-stage | <0.0001 | 0.4023 |
| **NCSLC subtype** (ADC *vs* SCC) |  |  |
| survival time (months) | 0.0470 | 0.2187 |
| survival event (DCD vs SURV) | 0.0066 | 0.2958 |
| T-stage | 0.0470 | 0.2187 |
| **UICC-stage** ǂ (increasing) |  |  |
| survival time (months) | 0.0006 | -0.3339 |
| survival event (DCD vs SURV) | <0.0001 | 0.3989 |
| T-stage | <0.0001 | 0.5190 |
| M-stage | <0.0001 | 0.9377 |
| **Treatment** (chemo only *vs* all others) |  |  |
| Stage (1-2 vs 3-4) | <0.0001 | 0.4549 |
| T-stage (T2-3 vs T4) | 0.0001 | 0.4402 |
| M-stage | <0.0001 | 0.9078 |
| survival time (months) (<25 vs >25) | <0.0001 | 0.4865 |
| survival event (DCD vs SURV) | 0.0064 | 0.3228 |

* significant correlations only are shown.

Pearson's correlation coefficients (r), 95% confidence intervals; p-value (two-tailed).

ǂ Union Internationale Contre le Cancer.

SURV, surviving; DCD, deceased.

**Supplementary Table 3: Antibodies used in the study.**

| **Primary antibodies** | | | | | |
| --- | --- | --- | --- | --- | --- |
| **Target** | **Clone** | **Company** | **Validation** | **REF** | **Dilution** |
| 2B4 | Rabbit pAb | Abcam | IHC-P | ab196745 | 1/50 |
| BTLA | MIH26 | eBioscience | IHC | 14-5979 | 1/25 |
| CD20 | L26 | Ventana | IHC-P | 760-2531 | 1/150 |
| CD26 | A6H | Sigma | IHC | SAB4200230 | 1/50 |
| CD3 | CD3-12 | Abcam | IHC-P | ab11089 | 1/50 |
| CD39 | 17B5F11 | Novus Biologicals | IHC-P | NBP2-25223 | 1/50 |
| CD4 | Goat pAb | R&D systems | IHC-P | AF-379-NA | 1/100 |
| CD45 | 2B11&PD7/26 | DAKO | IHC-P | M0701 | 1/50 |
| CD56 | MEM-188 | Abcam | IHC-P | ab8233 | 1/25 |
| CD57 | TB01 | eBioscience | IHC-P | 14-0577 | 1/100 |
| CD68 | KP1 | DAKO | IHC-P | M0814 | 1/50 |
| CD73 | D7F9A | Cell Signalling | IHC-P | 13160 | 1/200 |
| CD8a | 4B11 | ThermoFisher | IHC-P | MA1-80231 | 1/50 |
| CTLA-4 | 14D3 | eBioscience | IHC-P | 14-1529 | 1/50 |
| GZMB | GrB-7 | DAKO | IHC-P | M7235 | 1/50 |
| HLA-DR | TAL.1B5 | DAKO | IHC-P | M0746 | 1/50 |
| IFN-γ | Rabbit pAb | Abcam | IHC-P | ab9657 | 1/50 |
| IFN-γ | Rabbit pAb | Abcam | IHC-P | ab25101 | 1/50 |
| IFN-γ | RMMG-1 | Abcam | IHC-P | ab24979 | 1/50 |
| Ki-67 | Rabbit mAb | Thermofisher | IHC-P | RM-9106-R7 | 1/200 |
| LAG-3 | T47-530 | BD Biosciences | NA | 565717 | 1/25 |
| LAIR-1 | lc12 | Abcam | IHC-P | ab14826 | 1/50 |
| PD-1 | NAT105 | Abcam | IHC-P | ab52587 | 1/100 |
| PD-L1 | MIH1 | eBioscience | IHC | 14-5983 | 1/25 |
| PNAd | MECA-79 | BD Biosciences | IHC-P | 553863 | 1/100 |
| TIGIT | 741182 | R&D systems | NA | MAB7898 | 1/50 |
| TIM-3 | Rabbit pAb | Abcam | IHC-P | ab185703 | 1/100 |
| Cytokeratin 8/18 | EP17/EP30 | DAKO | IHC-P | IR094 | 1/50 |
| **Secondary antibodies** | | | | | |
| **Host/Target/Fluorophore** | |  | **Company** | **REF** | **Dilution** |
| Goat anti-Rabbit IgG (H+L) Cross-Adsorbed, Alexa Fluor 405 | | | Thermofisher | A-31556 | 1/250 |
| Goat anti-Mouse IgM (Heavy chain) Cross-Adsorbed, Alexa Fluor 488 | | | Thermofisher | A-21042 | 1/250 |
| Goat anti-Mouse IgG2a Cross-Adsorbed, Alexa Fluor 488 | | | Thermofisher | A-21131 | 1/250 |
| Goat anti-Mouse IgG2b Cross-Adsorbed, Alexa Fluor 594 | | | Thermofisher | A-21145 | 1/250 |
| Donkey anti-Mouse IgG (H+L) Highly Cross-Adsorbed, Alexa Fluor 594 | | | Thermofisher | A-21203 | 1/250 |
| Donkey anti-Rabbit IgG (H+L) Highly Cross-Adsorbed, Alexa Fluor 594 | | | Thermofisher | A-21207 | 1/250 |
| Donkey anti-Rat IgG (H+L) Highly Cross-Adsorbed, Alexa Fluor 594 | | | Thermofisher | A-21209 | 1/250 |
| Goat anti-Mouse IgG1 Cross-Adsorbed, Alexa Fluor 647 | | | Thermofisher | A-21240 | 1/250 |
| Goat anti-Rat IgM Cross-Adsorbed, DyLight 594 | |  | Thermofisher | SA5-10012 | 1/250 |
| Donkey anti-Rabbit IgG (H+L) Highly Cross-Adsorbed, Alexa Fluor 647 | | | Thermofisher | A-31573 | 1/250 |
| Goat anti-Rabbit IgG (H+L) Cross-Adsorbed, Alexa Fluor 750 | | | Thermofisher | A-21039 | 1/250 |
| Goat Anti-Rat IgG H&L (Alexa Fluor® 750) | |  | Abcam | ab175751 | 1/250 |
| Goat anti-Mouse IgG (H+L) Cross-Adsorbed, Alexa Fluor 750 | | | Thermofisher | A-21039 | 1/250 |

*Company guarantee that antibody is validated for FFPE tissues (IHC or IHC-P), or reference of prior validation.

**Supplementary Table 4. Correlations of TMA ICP MFI with immunogenicity and clinical characteristics.**

| **Mean fluorescence intensity (MFI)** | **p-value*** | **r value** |
| --- | --- | --- |
| **Gender** (female *vs* male) |  |  |
| TIM-3 | 0.0140 | -0.2427 |
| CTLA-4 | 0.0213 | -0.2278 |
| **T-stage** (T2-3 *vs* T4) |  |  |
| PD-L1 | 0.0037 | 0.2846 |
| **CD45** (high *vs* low) |  |  |
| LAIR-1 | 0.0385 | -0.2053 |
| **Histological subtype** (ADC *vs* SCC) |  |  |
| CTLA-4 | 0.0440 | -0.2217 |
| **Survival event** (DCD vs SURV) |  |  |
| CD57 | 0.0135 | 0.2014 |
| **Survival time** (months) |  |  |
| TIGIT | 0.0343 | -0.2098 |

* significant correlations only are shown.

Pearson's correlation coefficients (r), 95% confidence intervals; p-value (two-tailed).Supplementary Table 5. Correlations of TMA IIC and ICP counts with immunogenicity and clinical characteristics.

| **ICP and CD3-ICP cell counts** | **p-value** | **r value** |
| --- | --- | --- |
| **CD45** (hi *vs* low) |  |  |
| CD3-PD-1 | 0.0075 | 0.2295 |
| CD3-PD-L1 | 0.0266 | 0.2077 |
| CD3-BTLA | 0.0056 | 0.2499 |
| CD3-LAG-3 | 0.0174 | 0.2127 |
| **CD3** (hi *vs* low) |  |  |
| CD26 | <0.0001 | 0.4047 |
| TIM-3 | 0.0063 | 0.2302 |
| BTLA | <0.0001 | 0.3590 |
| LAG-3 | <0.0001 | 0.4218 |
| **Gender** (female *vs* male) |  |  |
| TIM-3 | 0.0399 | -0.3631 |
| CD8 | 0.0315 | -0.3335 |
| IFN-G | 0.0002 | -0.5895 |
| CD4 | 0.0086 | -0.4421 |
| CD68 | 0.0028 | -0.4733 |
| HLA-DR | 0.0469 | 0.0462 |
| PNAd | 0.0017 | 0.9042 |
| **Smoking** (yes *vs* no) |  |  |
| CD3 | 0.0385 | -0.3504 |
| PNAd | 0.0498 | 0.6059 |
| **T-stage** (T2-3 *vs* T4) |  |  |
| CD26 | 0.0268 | -0.1773 |
| 2B4 | 0.0433 | -0.1620 |
| CD57 | 0.0019 | -0.2462 |
| BTLA | 0.0212 | -0.1990 |
| CD3-CD26 | 0.0331 | -0.1707 |
| CD3-2B4 | 0.0281 | -0.1759 |
| CD3-CD57 | 0.0008 | -0.2659 |
| CD3-TIM-3 | 0.0157 | -0.2083 |
| CD3-LAG-3 | 0.0010 | -0.2808 |
| CD3-PD-L1 | 0.0318 | -0.1907 |
| **Metastasis** (yes *vs* no) |  |  |
| CD20 | 0.0370 | -0.3335 |
| GZMB | 0.0365 | -0.3565 |
| **Histological subtype** (ADC *vs* SCC) | |  |
| TIM-3 | 0.0065 | -0.2484 |
| CD73 | 0.0099 | 0.2189 |
| CD3-CD73 | 0.0078 | 0.2255 |

* significant correlations only are shown.

Pearson's correlation coefficients (r), 95% confidence intervals; p-value (two-tailed).

**Supplementary Table 6. Primary validation of positive association of ICP expression with OS.**

|  | **LUAD*** | | | **LUSC*** | | |
| --- | --- | --- | --- | --- | --- | --- |
| **ICP** | **p-value** | **HR** | **95% CI** | **p-value** | **HR** | **95% CI** |
| BTLA | 0.0013 | 0.4122 | 0.2400-0.7047 | ns | -- | -- |
| HAVCR2 (TIM-3) | 0.0013 | 0.3704 | 0.2109-0.6826 | ns | -- | -- |
| CTLA4 | 0.0068 | 0.3283 | 0.1930-0.5832 | ns | -- | -- |
| TIGIT | 0.0137 | 0.5142 | 0.2995-0.8659 | ns | -- | -- |
| DPP4 (CD26) | 0.0151 | 0.5291 | 0.3204-0.8824 | -- | -- | -- |
| LAIR1 | 0.0160 | 0.4799 | 0.2786-0.8743 | ns | -- | -- |
| B3GAT1 (CD57) | 0.0217 | 0.5764 | 0.3506-0.9181 | ns | -- | -- |
| ENTPD1 (CD39) | 0.0290 | 0.5059 | 0.2985-0.8165 | ns | -- | -- |

***** TCGA LUAD (*n* = 504) and LUSC (*n* = 494) datasets.

p-value, Log-rank test; HR, hazard ratio (Log-rank), CI, confidence interval of ratio.

**Supplementary Table 7. Secondary validation of positive association of ICP expression with OS.**

***Note: 7 is split in half for initial manuscript submission***

|  | **ADC*** | | | **SCC*** | | | **NSCLC (ADC + SCC)*** | | |
| --- | --- | --- | --- | --- | --- | --- | --- | --- | --- |
| **ICP** | **p-value** | **HR** | **95% CI** | **p-value** | **HR** | **95% CI** | **p-value** | **HR** | **95% CI** |
| BTLAǂ | 1.70E-07 | 0.53 | 0.42-0.68 | 3.44E-02 | 0.70 | 0.51-0.98 | 2.40E-07 | 0.65 | 0.55-0.77 |
| ENTPD1 (CD39)ǂ | 4.40E-07 | 0.45 | 0.33-0.62 | 2.00E-02 | 0.69 | 0.50-0.94 | 8.10E-11 | 0.57 | 0.49-0.68 |
| CTLA4ǂ | 1.10E-07 | 0.43 | 0.31-0.59 | -- | -- | -- | 6.40E-09 | 0.58 | 0.48-0.70 |
| DPP4 (CD26)ǂ | 5.20E-06 | 0.57 | 0.45-0.73 | -- | -- | -- | 1.80E-14 | 0.60 | 0.52-0.68 |
| B3GAT1 (CD57)ǂ | 2.40E-05 | 0.53 | 0.39-0.71 | -- | -- | -- | 2.00E-05 | 0.72 | 0.62-0.84 |
| NT5E (CD73) | 3.90E-05 | 0.61 | 0.49-0.78 | -- | -- | -- | 4.40E-06 | 0.74 | 0.65-0.84 |
| HAVCR2 (TIM-3)ǂ | 7.40E-03 | 0.70 | 0.54-0.91 | -- | -- | -- | 2.20E-06 | 0.67 | 0.57-0.79 |
| CD274 (PD-L1) | 4.40E-02 | 0.77 | 0.59-0.99 | -- | -- | -- | 1.30E-03 | 0.75 | 0.63-0.90 |
| CD244 (2B4) | -- | -- | -- | -- | -- | -- | 9.20E-03 | 0.85 | 0.74-0.96 |
| LAG3 | -- | -- | -- | 4.50E-04 | 0.64 | 0.50-0.82 | -- | -- | -- |
| TIGIT | -- | -- | -- | 1.30E-02 | 0.66 | 0.47-0.92 | -- | -- | -- |
| LAIR-1 | -- | -- | -- | 1.80E-02 | 0.83 | 0.64-1.08 | -- | -- | -- |

|  | **breast cancer (BC)*** | | | **gastric cancer (GI)*** | | | **ovarian cancer (OV)*** | | |
| --- | --- | --- | --- | --- | --- | --- | --- | --- | --- |
| **ICP** | **p-value** | **HR** | **95% CI** | **p-value** | **HR** | **95% CI** | **p-value** | **HR** | **95% CI** |
| BTLAǂ | 6.30E-03 | 0.64 | 0.46-0.88 | -- | -- | -- | -- | -- | -- |
| ENTPD1 (CD39)ǂ | 8.40E-04 | 0.56 | 0.39-0.79 | -- | -- | -- | -- | -- | -- |
| CTLA4ǂ | 1.50E-06 | 0.44 | 0.32-0.62 | 7.60E-05 | 0.62 | 0.49-0.79 | -- | -- | -- |
| DPP4 (CD26)ǂ | -- | -- | -- | 8.20E-03 | 0.76 | 0.62-0.93 | -- | -- | -- |
| B3GAT1 (CD57)ǂ | -- | -- | -- | -- | -- | -- | 2.50E-02 | 0.85 | 0.74-0.98 |
| NT5E (CD73) | -- | -- | -- | 8.20E-05 | 0.71 | 0.60-0.84 | -- | -- | -- |
| HAVCR2 (TIM-3)ǂ | -- | -- | -- | -- | -- | -- | -- | -- | -- |
| CD274 (PD-L1) | 1.00E-03 | 0.58 | 0.42-0.81 | 1.30E-05 | 0.62 | 0.50-0.77 | -- | -- | -- |
| CD244 (2B4) | 7.00E-04 | 0.68 | 0.55-0.85 | 6.60E-03 | 0.78 | 0.66-0.93 | 4.50E-02 | 0.86 | 0.74-1.00 |
| LAG3 | -- | -- | -- | 2.30E-02 | 0.82 | 0.69-0.97 | -- | -- | -- |
| TIGIT | 0.0001 | 0.48 | 0.33-0.71 | -- | -- | -- | 5.00E-02 | 0.82 | 0.67-1.00 |
| LAIR-1 | -- | -- | -- | -- | -- | -- | -- | -- | -- |

*from GEO, TCGA and EGA datasets; ADC (*n* = 673), SCC (*n* = 271), NSCLC (*n* = 1145), BC (*n* = 626), GI (*n* = 631), and OV (*n* = 655).

ǂ instances where effects of ICPs on OS are consistent with those from TCGA LUAD dataset.

p-value, Log-rank test; HR, hazard ratio (Log-rank), CI, confidence interval of ratio.

**Supplementary Table 8. ICP RNA expression in normal vs. cancer tissues.**

|  | **NSCLC*** | | **breast cancer (BC)*†** | | **gastric cancer (GI)*†** | | **ovarian cancer (OV)*†** | |
| --- | --- | --- | --- | --- | --- | --- | --- | --- |
|  | **Fold-change*** | **P value*** | **Fold-change*** | **P value*** | **Fold-change*** | **P value*** | **Fold-change*** | **P value*** |
| **Increased in expression in tumors vs normal** | | |  |  |  |  |  |  |
| LAG3ǂ | 3.185 | 6.16E-22 | 1.720 | 1.71E-06 | -- | -- | -- | -- |
| TIGITǂ | 2.298 | 3.31E-17 | 2.289 | 5.67E-14 | 1.512 | 9.98E-04 | -- | -- |
| BTLAǂ | 1.903 | 6.05E-13 | -- | -- | -- | -- | -- | -- |
| HAVCR2 (TIM-3)ǂ | 1.844 | 8.15E-06 | 1.604 | 5.41E-10 | 10.533 | 4.77E-30 | 1.603 | 2.60E-03 |
| PDCD1 (PD-1)ǂ | 1.645 | 2.14E-04 | 3.900 | 9.21E-13 | -- | -- | -- | -- |
| CTLA4 | 1.645 | 2.49E-06 | 1.509 | 4.60E-05 | 1.304 | 3.38E-03 | -- | -- |
| LAIR1 | 1.300 | 4.06E-07 | 1.642 | 1.03E-07 | 1.094 | 3.69E-02 | 1.642 | 4.00E-02 |
| **Unchanged in expression in tumors vs normal** | | |  |  |  |  |  |  |
| ENTPD1 (CD39) | 1.095 | 1.19E-01 | 1.020 | 5.59E-01 | 0.842 | 2.44E-01 | -- | -- |
| B3GAT1 (CD57) | 1.063 | 3.76E-01 | 1.000 | 8.94E-01 | -- | -- | 1.000 | 8.20E-01 |
| DPP4 (CD26) | 0.916 | 4.40E-01 | -- | -- | -- | -- | 0.608 | 2.70E-01 |
| **Decreased in expression in tumors vs normal** | | |  |  |  |  |  |  |
| CD244 (2B4) | 0.578 | 2.56E-02 | -- | -- | -- | -- | -- | -- |
| NT5E (CD73) | 0.731 | 1.65E-05 | 0.596 | 7.60E-06 | -- | -- | 0.596 | 1.30E-02 |
| CD274 (PD-L1) | 0.488 | 1.32E-10 | -- | -- | -- | -- | -- | -- |

* from GEO, TCGA and EGA datasets; NSCLC lung tumors (*n* = 2435) vs. normal lung tissues (*n* = 86); BC lung tumors (*n* = 6547) vs. normal breast tissues (*n* = 76); GI lung tumors (*n* = 1065) vs. normal gastric tissues (*n* = 57); OV lung tumors (*n* = 1648) vs. normal ovarian tissues (*n* = 5).

fold-change p-value singificance of magnitude of change between tumor (T) and normal (N) tissues.

ǂ demarcates ICP having positive association with OS in TMA study.

† values genes in agreement with those found for NSCLC are listed.**Supplementary Table 9. Validation of increased positive association with OS by ICP-TIL combination.**

|  | **NSCLC*** | | |
| --- | --- | --- | --- |
|  | **p-value** | **HR** | **95% CI** |
| CD4 | 1.40E-03 | 1.23 | 1.08-1.40 |
| CD8 | 1.80E-01 | 0.91 | 0.79-1.05 |
| **ICP group with positive effect on OS** | | |  |
| ICP alone | 2.30E-15 | 0.46 | 0.38-0.56 |
| ICP-CD4 | 2.20E-14 | 0.53 | 0.44-0.62 |
| ICP-CD8 | 1.30E-14 | 0.47 | 0.39-0.58 |
| **ICP group with higher expression in T vs N** | | |  |
| ICP alone | 5.20E-04 | 0.75 | 0.63-0.88 |
| ICP-CD4 | 1.30E-04 | 0.71 | 0.59-0.85 |
| ICP-CD8 | 7.60E-06 | 0.67 | 0.57-0.80 |

* from GEO, TCGA and EGA datasets; NSCLC (*n* = 1145).

p-value, Log-rank test; HR, hazard ratio (Log-rank), CI, confidence interval of ratio.**Supplementary Table 10. Validation of effect of IIC expression on OS.**

|  | **ADC+SCC** | | | | |
| --- | --- | --- | --- | --- | --- |
|  | **OS*** | | | **T vs N†** | |
| **IIC and effector** | **p-value** | **HR** | **95% CI** | **fold-change** | **p-value** |
| **higher expression in tumor relative to normal** | | | |  |  |
| CD45 (PRPTC) | 3.40E-03 | 0.82 | 0.72-0.95 | 2.423 | 2.73E-09 |
| Ki-67 (MKI67) | 2.60E-13 | 1.6 | 1.41-1.82 | 1.922 | 3.08E-09 |
| CD45-Ki-67 | 1.90E-02 | 1.19 | 1.03-1.37 | NA | NA |
| CD3-Ki-67 | 2.30E-01 | 1.09 | 0.95-1.24 | NA | NA |
| CD3 | 3.50E-03 | 0.81 | 0.71-0.93 | NA | NA |
| CD4 | 1.40E-03 | 1.23 | 1.08-1.40 | 1.031 | 1.40E-02 |
| CD8 | 1.80E-01 | 0.91 | 0.79-1.05 | 1.269 | 5.51E-07 |
| CD20 | 5.20E-03 | 0.82 | 0.71-0.94 | 1.956 | 3.48E-09 |
| IFNG | 1.30E-03 | 1.24 | 1.09-1.41 | 1.222 | 3.97E-03 |
| GZMB | 2.10E-04 | 1.33 | 1.14-1.54 | 1.798 | 7.05E-04 |
| HLA-DR | 8.40E-09 | 0.69 | 0.60-0.78 | NA | NA |
| **unchanged expression in tumor relative to normal** | | | |  |  |
| CD56 | 7.50E-03 | 0.83 | 0.72-0.95 | 0.793 | 6.44E-01 |
| CD68 | 8.50E-03 | 1.19 | 1.05-1.36 | 0.764 | 3.04E-01 |
| **lower expression in tumor relative to normal** | | | |  |  |
| PNAd (NTAN1) | 7.30E-06 | 0.71 | 0.61-0.83 | 0.554 | 5.69E-25 |

* from GEO, TCGA and EGA datasets; NSCLC (*n* =1926).

† lung tumors (T) (*n* = 2435) vs. normal lung tissues (N) (*n* = 86).

NA, where tumor vs normal tissue (i.e., T vs N) RNA expression of multiple genes cannot be assessed.

T vs N: p-value singificance of magnitude of change between tumor (T) and normal (N) tissues.

OS: p-value, Log-rank test; HR, hazard ratio (Log-rank), CI, confidence interval of ratio.**Supplementary Table 11. Chromosomal locations of profiled ICP.**

| **Gene** | **Chromosome** | **Location** | **Position** |
| --- | --- | --- | --- |
| CD244 (2B4) | 1 | NC_000001.11 | 160830160..160862902 |
| PDCD1 (PD-1) | 2 | NC_000002.12 | 241849881..241858908 |
| CTLA4 | 2 | NC_000002.12 | 203867788..203873960 |
| DPP4 (CD26) | 2 | NC_000002.12 | 161992241..162074542 |
| TIGIT | 3 | NC_000003.12 | 114293986..114310288 |
| BTLA | 3 | NC_000003.12 | 112458790..112499756 |
| HAVCR2 (TIM-3) | 5 | NC_000005.10 | 157085832..157109237 |
| CD274 (PD-L1) | 9 | NC_000009.12 | 5450503..5470567 |
| NT5E (CD73) | 9 | NC_000075.6 | 88327609..88372089 |
| ENTPD1 (CD39) | 10 | NC_000010.11 | 95710901..95877266 |
| B3GAT1 (CD57) | 11 | NC_000011.10 | 134378504..134411986 |
| LAG3 | 12 | NC_000012.12 | 6772483..6778455 |
| LAIR1 | 19 | NC_000019.10 | 54353624..54370556 |

Chromosomal locations of ICPs from <https://www.ncbi.nlm.nih.gov/gene>**Supplementary Table 12. Association of TMA ICP combinations from MP-IF panels with OS.**

| ICP and CD3-ICP combinations | p-value | HR | 95% CI |
| --- | --- | --- | --- |
| TIM-3-CD26 | 0.0061 | 0.4691 | 0.2192-0.7308 |
| TIM-3-CD39 | 0.1039 | 0.7303 | 0.4799-1.0460 |
| TIM-3-CTLA4 | 0.4779 | 1.159 | 0.7611-1.8270 |
| TIM-3-PD-L1 | 0.0056 | 0.3908 | 0.1961-0.7243 |
| TIM-3-PD-1 | 0.0229 | 0.4413 | 0.2057-0.8724 |
| CD26-CD39 | 0.0022 | 0.4997 | 0.3404-0.7664 |
| CD26-CTLA-4 | 0.5017 | 0.873 | 0.5764-1.2970 |
| CD39-CTLA4 | 0.2085 | 1.268 | 0.8770-1.9520 |
| PD-1-2B4 | 0.0017 | 0.3827 | 0.1924-0.6454 |
| PD-1-CD57 | 0.0169 | 0.4731 | 0.2371-0.8325 |
| 2B4-CD57 | 0.3236 | 0.7458 | 0.3915-1.3320 |
| TIGIT-LAIR-1 | 0.1694 | 0.6676 | 0.3601-1.1610 |
| TIGIT-CD73 | 0.7962 | 0.9402 | 0.5729-1.5240 |
| LAIR-1-CD73 | 0.0948 | 0.5992 | 0.3481-1.0540 |
| TIM-3-BTLA | 0.0022 | 0.4284 | 0.1936-0.6629 |
| TIM-3-LAG-3 | 0.0032 | 0.426 | 0.2069-0.6894 |
| TIM-3-CD26-CD39 | 0.0139 | 0.5292 | 0.2672-0.8988 |
| TIM-3-CD26-CTLA4 | 0.2755 | 0.7554 | 0.4518-1.2330 |
| TIM-3-CD39-CTLA4 | 0.5099 | 0.8775 | 0.5818-1.2950 |
| CD26-CD39-CTLA4 | 0.6680 | 0.8856 | 0.5083-1.5430 |
| PD-1-2B4-CD57 | 0.0093 | 0.3729 | 0.1949-0.7533 |
| TIGIT-LAIR-1-CD73 | 0.1682 | 0.6442 | 0.3602-1.1580 |
| TIM-3-BTLA-LAG-3 | 0.0018 | 0.4007 | 0.2029-0.6638 |
| TIM-3-CD26-CD39-CTLA4 | 0.2592 | 0.7963 | 0.5234-1.1700 |
| CD3-TIM-3-CD26-CD39 | 0.0051 | 0.4465 | 0.2120-0.7219 |
| CD3-PD-1-2B4-CD57 | 0.0080 | 0.4183 | 0.2214-0.7906 |
| CD3-TIM-3-BTLA-LAG-3 | 0.0033 | 0.4229 | 0.1908-0.6807 |

OS: p-value, Log-rank test; HR, hazard ratio (Log-rank), CI, confidence interval of ratio.**Supplementary Table 13. Figure 5A correlogram common ICP groupings.**

| **TCGA LUSC and LUAD** | | |
| --- | --- | --- |
| **group 1** | **group 2** | **outliers** |
| BTLA* | CD73 | PD-L1 |
| TIM-3* | CD26* | CD39* |
| LAG-3 |  | CD57* |
| CTLA-4* |  |  |
| TIGIT* |  |  |
| LAIR-1* |  |  |
| PD-1 |  |  |
| 2B4 |  |  |
| **TMA ICP** | | |
| **group 1** | **group 2** | **group 3** |
| BTLA | TIGIT | PD-L1 |
| TIM-3* | CD73 | LAIR-1 |
| LAG-3 | CD57 | PD-1 |
| CD26 | 2B4 |  |
| CTLA4 |  |  |
| CD39 |  |  |
| **TMA CD3-ICP** | | |
| **group 1** | **group 2** | **group 3** |
| BTLA* | TIGIT* | CTLA-4 |
| TIM-3* | CD73 | CD39 |
| LAG-3* | CD57 | CD26 |
| PD-L1 | 2B4 | PD-1* |
| LAIR-1 |  |  |

* denotes independent positive association to OS.

underlined denotes conserved group across sets.

**Supplementary Table 14: Fig S7 PC1 and PC2 groups positively associated with OS.**

| **TCGA** | | **TMA** | |
| --- | --- | --- | --- |
| **LUAD PC1** | **LUSC PC2** | **ICP PC1** | **CD3-ICP PC1** |
| BTLA* | BTLA | BTLA | BTLA* |
| LAG-3 | LAG-3 | LAG-3 | LAG-3* |
| PD-1 | PD-1 | PD-1 | PD-1* |
|  | PD-L1 | PD-L1 | PD-L1 |
| 2B4 | 2B4 | TIM-3* | TIM-3* |
| CTLA-4* | CTLA-4 | CD26 | CD26 |
| TIGIT* | TIGIT | CD39 | CD39 |
|  | CD57 |  |  |

* denotes independent positive association to OS.

underlined denotes conserved group across sets.**Supplementary Table 15. ICP-interactors having effects on K-M and modulated in their expression.**

| **Partner**  **Uniprot**  **ID** | **Partner Symbol** | **# ICP-interactions** | **K-M prognosis (pos/neg)** | **K-M**  **P-values** | **T vs N** | **T vs N**  **P-value** |
| --- | --- | --- | --- | --- | --- | --- |
| P09326 | CD48 | 7 | pos | 0.061 | T | 2.56E-05 |
| Q9NQ25 | SLAMF7 | 7 | pos | 0.00054 | T | 4.20E-33 |
| P06729 | CD2 | 6 | pos | 2.00E-10 | N | 2.39E-15 |
| Q9NZQ7 | CD274 | 6 | pos | 0.0013 | N | 1.32E-10 |
| Q5ZPR3 | CD276 | 6 | neg | 9.00E-07 | T | 2.31E-08 |
| P01730 | CD4 | 6 | neg | 1.40E-03 | T | 1.40E-02 |
| P06241 | FYN | 6 | pos | 1.70E-10 | N | 3.21E-06 |
| P06239 | LCK | 6 | pos | 7.60E-09 | T | 2.48E-17 |
| Q9HBG7 | LY9 | 6 | pos | 1.00E-09 | T | 2.51E-12 |
| Q9BQ51 | PDCD1LG2 | 6 | neg | 5.30E-05 | N | 1.49E-14 |
| Q15235 | PSG6 | 6 | neg | 1.10E-05 | N | 4.35E-17 |
| Q9Y279 | VSIG4 | 6 | pos | 4.30E-02 | T | 2.76E-03 |
| Q6UX41 | BTNL8 | 5 | neg | 1.70E-02 | N | 2.01E-03 |
| P10747 | CD28 | 5 | pos | 9.30E-11 | T | 7.20E-05 |
| Q8TDQ1 | CD300LF | 5 | pos | 3.40E-08 | T | 4.33E-04 |
| P07766 | CD3E | 5 | neg | 1.20E-07 | N | 2.33E-04 |
| P25942 | CD40 | 5 | pos | 6.10E-06 | T | 1.01E-14 |
| P10966 | CD8B | 5 | pos | 5.10E-03 | T | 7.44E-04 |
| Q14002 | CEACAM7 | 5 | neg | 2.50E-02 | N | 1.32E-02 |
| P78310 | CXADR | 5 | pos | 1.80E-06 | T | 2.63E-06 |
| P01891 | HLA-A | 5 | neg | 5.70E-05 | T | 2.18E-13 |
| P06340 | HLA-DOA | 5 | pos | 5.30E-03 | T | 6.06E-05 |
| P16871 | IL7R | 5 | pos | 1.10E-15 | N | 5.06E-18 |
| Q92835 | INPP5D | 5 | pos | 1.20E-05 | N | 2.34E-02 |
| P11215 | ITGAM | 5 | pos | 1.80E-07 | T | 2.69E-03 |
| Q8N423 | LILRB2 | 5 | neg | 2.50E-02 | T | 2.03E-02 |
| Q9UDY8 | MALT1 | 5 | pos | 2.10E-05 | N | 4.28E-12 |
| Q16653 | MOG | 5 | neg | 2.90E-03 | N | 1.73E-04 |
| Q06124 | PTPN11 | 5 | pos | 2.50E-07 | N | 2.54E-31 |
| Q9BX59 | TAPBPL | 5 | pos | 1.30E-03 | T | 4.59E-20 |
| Q9NZC2 | TREM2 | 5 | neg | 1.30E-02 | T | 6.53E-37 |
| P15498 | VAV1 | 5 | pos | 3.10E-09 | T | 1.73E-05 |
| Q7Z6A9 | BTLA | 4 | pos | 2.40E-07 | T | 6.05E-13 |

*Preview of table containing 307 ICP-interactors with effects on prognosis and modulation of RNA expression in tumors (T) relative to normal tissues.*

* from GEO, TCGA and EGA datasets; NSCLC (*n* =1926).

† lung tumors (T) (*n* = 2435) vs. normal lung tissues (N) (*n* = 86).

T vs N: p-value singificance of magnitude of change between tumor (T) and normal (N) tissues.

OS: p-value, Log-rank test; HR, hazard ratio (Log-rank), CI, confidence interval of ratio.

**Supplementary Table 16. ICP-ICP interactors from IID.**

| **Query Symbol** | **# of interacting ICP** | **ICP-ICP interactors** | **tot # of interactors** |
| --- | --- | --- | --- |
| BTLA* | 5 | PD-L1, TIM-3, TIGIT, CTLA-4, LAIR-1 | 262 |
| PD-L1* | 5 | BTLA, TIM-3, TIGIT, CTLA-4, PD-1 | 265 |
| TIM-3* | 5 | BTLA, PD-L1, TIGIT, LAIR-1, 2B4, | 170 |
| TIGIT | 4 | BTLA, PD-L1, TIM-3, CTLA-4 | 361 |
| CTLA-4* | 3 | BTLA, PD-L1, TIGIT | 169 |
| LAIR-1 | 2 | BTLA, TIM-3 | 150 |
| 2B4* | 1 | TIM-3 | 92 |
| CD39 | 1 | CD73 | 54 |
| CD73* | 1 | CD39 | 93 |
| PD-1 | 1 | PD-L1 | 16 |
| CD57 | 0 | 0 | 22 |
| CD26 | 0 | 0 | 81 |
| LAG-3 | 0 | 0 | 15 |

* ICP having positive effect on OS.

# of interactors calculated from IID.

**Supplementary Table Table S17: Positive T cell functions of selected NSCLC patient stratifying ICPs**

| **ICP** | **Function** |
| --- | --- |
| TIM-3 | its targeting leads to autoimmune diseases^1^  is important for healthy pregnancies^2^  in NSCLC, is expressed on tumor cells and TILs^3^  its mutation is associated with lowered survival rates^4^  is more highly expressed in NSCLC than in normal tissues^5^  its expression on CD4^+^, but not CD8^+^ T cells, correlates with advanced NSCLC and metastasis^5^  its expression in NSCLC is linked to stronger immune responses^3^  its expression is increased and sustained in T cells from stimulation of NSCLC PBMCs for ACT^6^  is frequently expressed by CD8^+^CD103^+^ TILs correlating with improved NSCLC survival^7^  its absence reduces CD8^+^ T cell responses to infection, and correlates with decreased IFN-γ production and degranulation^8^ |
| LAG-3 | its absence leads to increased antigen-induced cell death and defects on T cell expansion^9^  its stimulation of APC, may induce cytokine production and expression of costimulatory ligands for T cell survival^9^  its expression identifies an HIV-antigen specific CD8^+^ T cell population, its levels are highest in individuals with lower viral load^10^  it was found to be highly increased in non-naïve (GZMB, IFN-γ, TNF-α, IL-2) CD8^+^ T cells in a study proving that ICP are not exhaustion markers^11^  it can compensate for lack of PD-1 signaling, mediates CD8 impairment in lungs, and contributes to protection from immunopathology during viral clearance^12^ |
| BTLA | its decrease on lymphocytes is associated to declining lung function^13^  its absence results in severe mucosal inflammation in the gut and lung, autoimmune-like disease, impaired bacterial-immunity^12^  its absence results in rejection of MHC-mismatched cardiac transplants^14^  its deficiency on T cells leads to poorer survival in a graft-vs-host disease model^15^  its absence causes hypergammaglobulinemia, hepatitis-like disease, and significantly reduced survival rates in mice^16^ |
| TIGIT | it selectively suppresses proinflammatory Th1 and Th17 responses but spares that of Th2^17^  its loss in mice results in hyperproliferative T cell responses and increased susceptibility to autoimmunity^18^  it marks dysfunctional CD8^+^ Tregs but not effector CD8^+^ T cells^19,20^  its expression in melanoma patients identifies the antigen-specific CD8^+^ TIL fraction^21^  is expressed by NSCLC TILs displaying a recently activated, non-exhausted phenotype^22^ |

**Supplementary Table 17 References**

1. Sanchez-Fueyo, A.*, et al.* Tim-3 inhibits T helper type 1-mediated auto- and alloimmune responses and promotes immunological tolerance. *Nature immunology* **4**, 1093-1101 (2003).

2. Meggyes, M.*, et al.* Peripheral blood TIM-3 positive NK and CD8+ T cells throughout pregnancy: TIM-3/galectin-9 interaction and its possible role during pregnancy. *PloS one* **9**, e92371 (2014).

3. Zhuang, X.*, et al.* Ectopic expression of TIM-3 in lung cancers: a potential independent prognostic factor for patients with NSCLC. *Am J Clin Pathol* **137**, 978-985 (2012).

4. Bai, J.*, et al.* T-cell immunoglobulin- and mucin-domain-containing molecule 3 gene polymorphisms and prognosis of non-small-cell lung cancer. *Tumour Biol* **34**, 805-809 (2013).

5. Gao, X.*, et al.* TIM-3 expression characterizes regulatory T cells in tumor tissues and is associated with lung cancer progression. *PloS one* **7**, e30676 (2012).

6. Zhang, L.*, et al.* Profiling the dynamic expression of checkpoint molecules on cytokine-induced killer cells from non-small-cell lung cancer patients. *Oncotarget* (2016).

7. Djenidi, F.*, et al.* CD8+CD103+ tumor-infiltrating lymphocytes are tumor-specific tissue-resident memory T cells and a prognostic factor for survival in lung cancer patients. *Journal of immunology (Baltimore, Md. : 1950)* **194**, 3475-3486 (2015).

8. Gorman, J.V.*, et al.* Tim-3 directly enhances CD8 T cell responses to acute Listeria monocytogenes infection. *Journal of immunology (Baltimore, Md. : 1950)* **192**, 3133-3142 (2014).

9. Workman, C.J. & Vignali, D.A. The CD4-related molecule, LAG-3 (CD223), regulates the expansion of activated T cells. *Eur J Immunol* **33**, 970-979 (2003).

10. Pena, J., Jones, N.G., Bousheri, S., Bangsberg, D.R. & Cao, H. Lymphocyte activation gene-3 expression defines a discrete subset of HIV-specific CD8+ T cells that is associated with lower viral load. *AIDS Res Hum Retroviruses* **30**, 535-541 (2014).

11. Legat, A., Speiser, D.E., Pircher, H., Zehn, D. & Fuertes Marraco, S.A. Inhibitory Receptor Expression Depends More Dominantly on Differentiation and Activation than "Exhaustion" of Human CD8 T Cells. *Frontiers in immunology* **4**, 455 (2013).

12. Erickson, J.J., Rogers, M.C., Tollefson, S.J., Boyd, K.L. & Williams, J.V. Multiple Inhibitory Pathways Contribute to Lung CD8+ T Cell Impairment and Protect against Immunopathology during Acute Viral Respiratory Infection. *Journal of immunology (Baltimore, Md. : 1950)* **197**, 233-243 (2016).

13. Liu, J.*, et al.* [The decline in lung function is associated with a decrease in the number of BTLA(+) lymphocytes and regulatory T cells in patients with rheumatism]. *Xi Bao Yu Fen Zi Mian Yi Xue Za Zhi* **30**, 639-642 (2014).

14. Tao, R.*, et al.* Differential effects of B and T lymphocyte attenuator and programmed death-1 on acceptance of partially versus fully MHC-mismatched cardiac allografts. *Journal of immunology (Baltimore, Md. : 1950)* **175**, 5774-5782 (2005).

15. Hurchla, M.A., Sedy, J.R. & Murphy, K.M. Unexpected role of B and T lymphocyte attenuator in sustaining cell survival during chronic allostimulation. *Journal of immunology (Baltimore, Md. : 1950)* **178**, 6073-6082 (2007).

16. Oya, Y.*, et al.* Development of autoimmune hepatitis-like disease and production of autoantibodies to nuclear antigens in mice lacking B and T lymphocyte attenuator. *Arthritis Rheum* **58**, 2498-2510 (2008).

17. Joller, N.*, et al.* Treg cells expressing the coinhibitory molecule TIGIT selectively inhibit proinflammatory Th1 and Th17 cell responses. *Immunity* **40**, 569-581 (2014).

18. Joller, N.*, et al.* Cutting edge: TIGIT has T cell-intrinsic inhibitory functions. *Journal of immunology (Baltimore, Md. : 1950)* **186**, 1338-1342 (2011).

19. Kurtulus, S.*, et al.* TIGIT predominantly regulates the immune response via regulatory T cells. *The Journal of clinical investigation* **125**, 4053-4062 (2015).

20. Johnston, R.J.*, et al.* The immunoreceptor TIGIT regulates antitumor and antiviral CD8(+) T cell effector function. *Cancer cell* **26**, 923-937 (2014).

21. Chauvin, J.M.*, et al.* TIGIT and PD-1 impair tumor antigen-specific CD8(+) T cells in melanoma patients. *The Journal of clinical investigation* **125**, 2046-2058 (2015).

22. Tassi, E.*, et al.* Early effector T lymphocytes coexpress multiple inhibitory receptors in primary non-small cell lung cancer. *Cancer research* (2016).
